# The Effect of Climate Variables, Soil Characteristics, and Peanut Cultivars on the Rhizobial Bacteria Community

**DOI:** 10.3390/microorganisms13040926

**Published:** 2025-04-17

**Authors:** Juan Li, Zhong-De Yang, En-Tao Wang, Li-Qin Sun, Yan Li

**Affiliations:** 1 Yantai Key Laboratory of Characteristic Agricultural Bioresource Conservation & Germplasm Innovative Utilization, College of Life Sciences, Yantai University, Yantai 264005, China; 2 Departamento de Microbiología, Escuela Nacional de Ciencias Biológicas, Instituto Politécnico Nacional, Ciudad de México 11340, Mexico

**Keywords:** peanut, *Bradyrhizobium*, diversity, distribution, biogeography

## Abstract

Peanuts are widely cultivated across the world; however, peanut’s rhizobial community and the determinant factors of their composition are still to be elucidated. This study investigates the biogeography and determinant soil environmental factors for peanut rhizobia. A total of 1001 rhizobial isolates were obtained from the peanut root nodules, mainly belonging to two cultivars (X9 and M6) cultivated in 20 sampling sites across China. According to *recA* sequence analysis, all the isolates were classified as 84 haplotypes, and a representative strain for each haplotype was randomly selected to perform subsequent analyses. Based on multilocus sequence analysis (MLSA) of housekeeping genes *dnaK*, *glnII*, *gyrB*, *recA*, and *rpoB*, all the representative strains were classified as 42 genospecies in the genus *Bradyrhizobium*, including 12 effectively published and 30 undefined genospecies. Strains belonging to six genospecies were predominant (>5%), including *B. ottawaense*, *B. liaoningense*, *B. yuanmingense*, *Bradyrhizobium* sp. XXIX, *B. guangdongense*, and *B. nanningense*. However, only a single isolate was obtained for 15 genospecies. The diversity indices of peanut rhizobia distributed in South China are obviously higher than those in North China, but no obvious peanut cultivar selection for rhizobial genospecies was found. Correlation analyses indicated that the community composition of peanut rhizobia was mainly affected by MAP, MAT, soil AP, and pH. Nodulation tests indicated that the 79 representative strains belonging to 37 genospecies with both *nodC* and *nifH* could perform nitrogen-fixing symbiosis with peanuts. This study revealed the great diversity and varied composition of communities of peanut rhizobia in different geographic regions across China.

## 1. Introduction

Peanut or ground nut (*Arachis hypogaea* L.) is an annual oil and grain legume crop that originated in South America and is now cultivated throughout the world, mainly in tropical and subtropical agro-climatic areas of Asia, Africa, and the Americas [1,2]. As the second most widely cultivated grain legume in the world after soybean, it has important applications in the oil, culinary, food, and pharmaceutical industries [3]. Peanut was introduced and cultivated in China about 500 years ago [4], and now China is the largest peanut producer in the world, with more than 16 million tons of production per year and accounting for 45% of the total production across the world (http://faostat3.fao.org/, accessed on 12 June 2024). Meanwhile, peanut accounts for >46% of the total output of all oil crops in China [5], and China is also a major consumer of peanut oil, consuming 54.81% of the world’s total domestic consumption [6]. As a member of the tribe *Aeschynomeneae*, the symbiosis of peanut and the partner rhizobia was established through a more primitive mold of “crack entry” [7,8,9]. Through this kind of symbiotic nitrogen fixation process, the two symbiotic partners provide a more economical and ecological nitrogen source in the field and also significantly increase the peanut yield [10].

Peanut is thought to be a promiscuous host for rhizobia due to its nodulates with diverse rhizobial species. Although minor fast-growing rhizobia belonging to genera *Rhizobium* and *Sinorhizobium* (*Ensifer*) were obtained from Morocco, Argentine, South Africa, and China as well [11,12,13,14], the main peanut nodulation symbionts isolated across the world were members in the genus *Bradyrhizobium* [4,11,12,15,16,17]. Furthermore, the peanut-nodulating *Bradyrhizobium* strains have been affiliated with more than 30 genospecies, including 17 defined species (*B. arachidis*, *B. diazoefficiens*, *B. elkanii*, *B. ganzhouense*, *B. guangdongense*, *B. guangxiense*, *B. guangzhouense*, *B. iriomotense*, *B. japonicum*, *B. kavangense*, *B. lablabi*, *B. liaoningense*, *B. nanningense*, *B. ottawaense*, *B. subterraneum*, *B. yuanmingense*, and *B. zhanjiangense*) [18,19,20,21]. In some of these species, strains also nodulating with other legumes, such as soybean, *Chamaecrista mimosoides*, *Lablab purpureus*, and *Vigna minima* [22,23,24,25], are also included. Furthermore, 14 candidate new (geno)species were also reported in several previous studies [15,26].

Each rhizobial species usually owns its most suitable environment, which results in their biogeography distribution patterns [24]. Previously, soil environmental variables such as soil pH have been reported to be the main factor affecting the distribution of rhizobia and further determine *Glycine max* symbiosis with *Sinorhizobium* spp. in alkaline soils or *Bradyrhizobium* spp. in acid soils [23], and climate variables such as mean annual precipitation and mean annual temperature also correlated with the soil microbial community [27]. In addition, the nodulating rhizobia could also be determined using the host’s preferable selection; for example, the *R* gene of soybean could restrict nodulation by specific rhizobial strains of *Bradyrhizobium japonicum* or *Sinorhizobium fredii* [28]. Different common bean and clover varieties showed obviously preferable selection for rhizobial species [29,30]. In relation to the double selection from soil conditions and host plants, peanut rhizobia also showed clear biogeographic distribution patterns; for example, *B. liaoningense* and *B. ottawaense* were dominant in Shandong Province, China [17], whereas *B. guangdongense* is dominant in Henan and Guangdong Provinces [15,26,31]. However, the biogeographic distribution patterns and the main determinant factors, such as soil environmental factors or cultivar selection for peanut rhizobia, are still to be studied. To assess the contributions of soil factors and host varieties to the biogeography of peanut rhizobia, we collected peanut root nodules in this study (mainly from two peanut cultivars: Xuhua 9 (X9) and Minhua 6 (M6) [32]) in 20 sites across China. X9 is an irregular peanut bred by the Institute of Agricultural Sciences of Xuzhou [32]. M6 is a Spanish peanut bred by the College of Crop Science, Fujian Agriculture and Forestry University [32]. The seed of X9 presents a high fat content, and M9 contains high protein. By comparing peanut rhizobia from the same cultivar across China and the rhizobia from different cultivars in the same site, we aim to (1) uncover the geographic distribution, bacterial diversity, and community structure of peanut rhizobia across China and (2) compare and analyze the contribution of soil characteristics and cultivar selection for rhizobial distribution.

## 2. Material and Methods

### 2.1. Soil and Nodule Sampling

**Figure 1:**
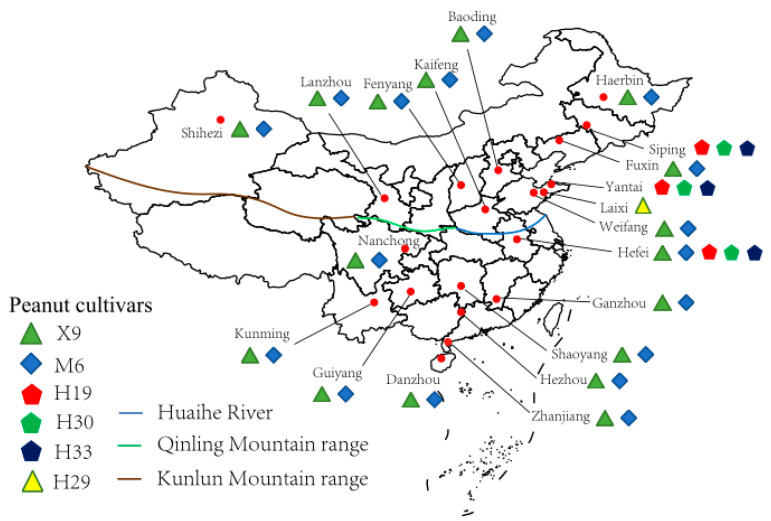
Map of the sampling sites (●) across China. The map was created using R 4.3.2, and the sampling sites were added according to GPS records. Root nodules from the different peanut cultivars in different sites are labeled.

Peanut root nodules were collected from 20 sampling sites across China from 2015 through 2018 (Figure 1, Table 1 and Table S1). All the nodules were sampled at the early kernel stage of peanut. A total of 6 peanut cultivars were referred during the sampling, in which Xuhua 9 (X9) and Minhua 6 (M6) were recorded in 16 of the 20 sampling sites; Huayu 19 (H19), Huayu 30 (H30), and Huayu 33 (H33) were the only cultivars in two sampling sites (Siping and Yantai); and all the five cultivars mentioned above were planted in the sampling site Hefei. At the same time, the remaining cultivar, Huayu 29 (H29), was only sampled in Laixi. At each sampling site, 5–10 randomly selected peanut plants of each cultivar were uprooted, and 30–50 root nodules from each plant were carefully picked and transferred directly to laboratory or put in a sterilized tube filled with silica gel particles for transfer to the laboratory for the subsequent bacterial isolation (Table S2). In the meantime, soil samples were also collected from root zone (0–20 cm depth) of each plant, which were mixed in a sterile bag at a ratio of similar volume and preserved on ice for transfer to the laboratory. Then, the soil samples were air-dried for physiochemical characterization.

### 2.2. Rhizobial Isolation and Soil Environmental Characterization

For rhizobial isolation, 50–100 uniformly sized round dehydrated nodules (in sterilized 0.85% NaCl solution at 4 °C for 6 h) or fresh nodules from per cultivar/per site were mixed and immersed in 95% ethanol for 30 s to eliminate nodule surface tension and then transferred to a sterilized beaker for surface sterilization (3% NaClO for 3 min). Then, the nodules were washed with sterilized deionized water five times, and each nodule was transferred to a 1.5 mL sterilized tube, crushed into juice, and inoculated on yeast mannitol agar (YMA) plates, as described previously [33]. To evaluate the efficiency of the sterilization method, 100 µL water from the last rinse was also plated on YMA plates. The inoculated plates were incubated at 28 °C for 7–40 days, carefully checked every two days, and a single colony was randomly selected from each plate and purified via repeated streaking on YMA plates. The purified isolates were preserved in YM broth supplied with 20% (*v*/*v*) glycerol at −80 °C.

For soil physiochemical analyses, all the soil samples were air-dried and sieved using a 2 mm mesh. The physiochemical characteristics were determined using the corresponding standard protocols: soil pH was determined with a soil–water (1:2.5 *w*/*v*) suspension using a pH meter [34], the concentration of available nitrogen (AN) was determined by means of quantifying the alkali-hydrolyzable nitrogen [35], the content of available phosphorous (AP) was determined using the Olsen’s Method via a colorimetry method [36], the content of available potassium (AK) was measured by means of NH_4_OAc extraction and the flame photometer method at a wavelength of 767 nm [37], the concentration of total nitrogen (TN) measured using titration method with standard acid [35], and the organic carbon (OC) content in soil was determined using wet oxidation method with K_2_Cr_2_O_7_-concentrated H_2_SO_4_ [36]. And the electrical conductivity (EC) was established for the soil filtrate using a WTW/LF-330 conductivity meter [38]. The climate variables, including mean annual precipitation (MAP) and mean annual temperature (MAT) for each sampling site, were downloaded from the WorldClim database (https://www.worldclim.org/, accessed on 10 August 2024). The temperature seasonality (TS) was calculated using the ratio of the standard deviation of the monthly mean temperatures to the mean of the monthly temperatures. And the precipitation seasonality (PS) was calculated using the ratio of the standard deviation of the monthly total precipitation to the mean monthly total precipitation [27].

### 2.3. PCR Amplification and Sequencing

The genomic DNA of each isolate was extracted using the TIANGE genomic DNA extraction kit for bacteria (TIANGEN, Beijing, China). All the DNA samples were used as template to amplify the *recA* genes by using the primer pair recA41F/recA640R and the corresponding PCR protocol [39]. The amplicons were sequenced directly with the commercial service at Beijing AuGCT DNA-SYN Biotechnology Co., Ltd. (Beijing, China) using Sanger methods [40]. The obtained sequences were aligned using MUSCLE program integrated in MEGA 7.0 [41]. All the sequences were classified into *recA* haplotypes as previously reported by using DNASP v5 [42,43,44]. Then, a representative strain for each *recA* haplotype was randomly selected for further study.

### 2.4. Phylogenetic Analysis of Housekeeping Genes

The other four housekeeping genes, including *dnaK*, *glnII*, *gyrB*, and *rpoB* of each representative strain, were amplified using the classic primer pairs glnII12F/glnII689R [45], gryB343F/gryB1043R [46], TsdnaK3/TsdnaK2 [45], and rpoB454F/rpoB1364R [47], as well as the corresponding protocols, respectively. All the amplicons were sequenced directly as described for *recA* sequencing. Each obtained sequence was aligned by using online BLAST program 2.16.0 of NCBI web (https://www.ncbi.nlm.nih.gov/, accessed on 20 February 2024), and the related corresponding reference sequences of type strains were downloaded.

Multilocus sequence analysis (MLSA) is well known for providing data with greater discriminatory ability than analysis using a single gene [48]. The 97.3% sequence similarity in MLSA was proposed as a threshold for rhizobium genospecies classification [44,48]. In this study, the MLSA was conducted using the concatenated housekeeping genes of *dnaK*, *glnII*, *gyrB*, *recA*, and *rpoB* sequences with a 97.3% sequence similarity to classify the representative strains into genospecies, as recommended in previous studies [17,48]. The sequence similarities between the obtained sequences in this study and those of reference-type strains extracted (blasted) from GenBank database were evaluated using MEGA 7.0 [41]. A neighbor-joining phylogenetic tree was reconstructed using the concatenated sequences by MEGA 7.0 based on Kimura 2-parameter model, and the topology was evaluated using the bootstrap method with 1000 replicates [41].

### 2.5. Phylogenetic Analyses for Symbiosis Genes and Nodulation Test for Representative Strains

*nodC* and *nifH* of all the type strains were amplified using pair primers of nodC-for540/nodC-rev1160 and nifH-F/nifH-R [45]. All the representative strains were applied to nodulation test as previously described [17,44]. The intact plump peanut (X9) seeds were surface sterilized in 3% (*w*/*v*) sodium hypochlorite solution for 5 min and then washed five times by using autoclaved deionized water. Then, the disposed seeds were germinated on water–agar plates (0.6%) in the dark at 28 °C for 72 h. For each representative strain, 1 mL of the desired suspension (OD600 = 0.2) in 0.85% (*w*/*v*) NaCl solution was inoculated into each seedling planted in sterilized vermiculite (irrigated with low nitrogen solution) in a Leonard jar, and this test was performed in triplicate [17]. Plants inoculated with 0.85% (*w*/*v*) NaCl solution were included as blank controls. Then, the inoculated seedlings were transferred to an automatic artificial greenhouse with a daylight illumination period of 12 h and were harvested at 45 days post-inoculation (dpi). The plants with dark green leaves and pink nodules were judged to have effective symbiosis [17]. The control plants presented yellow leaves without nodules.

### 2.6. Peanut Rhizobial Diversity and Correlation Analyses

The peanut rhizobial diversity, richness, and evenness of each sampling site were analyzed, and the ecological indexes of Shannon–Wiener index (*H′*), Simpson index (*D*), and Pielou index (*J*), as well as principal component analysis (PCA), were estimated by using the Vegan package (version 2.5-7) and pacman (v.0.5.1) on the R statistical language platform (version 4.1.1) [49]. The correlation between rhizobial genospecies (the genospecies abundance > 1% was selected) and soil characteristics was analyzed using CANOCO 5.0 [50]. One-way ANOVAs were selected to test the difference significance of diversity indices between different samples and the difference significance among each soil physiochemical characteristic from different sampling sites using Tukey’s test integrated in SPSS 27. And two-way MANOVA analyses were selected to test the difference significance of the interactive effect between peanut cultivar and rhizobium genospecies using SPSS 27; during the analyses, each dependent variable followed multivariate normal distribution through Mahalanobis test. The main effect of peanut cultivar, soil characteristics, and the interaction of peanut cultivar and rhizobium genospecies were evaluated using Tukey’s test.

## 3. Results

### 3.1. Soil Characteristics

The determined soil characteristics are presented in Table 1; the soil physiochemical characteristics were analyzed in triplicate, and each data piece consists of a mean and a standard deviation. The soil characteristics from different sampling sites showed significant differences. Soil pH values ranged from 4.92 ± 0.18 in Danzhou to 8.64 ± 0.20 in Fenyang. The EC values ranged from 52.80 ± 4.5 μs/cm in Siping to 288.0 ± 4.3 μs/cm in Lanzhou. The content of main mineral nutrients in the dry soil samples were as follows (mg/kg): 26.8 ± 3.6–141.2 ± 2.5 for AN, 10.1 ± 1.6–82.8 ± 1.3 for AP, 199.2 ± 5.1–437.0 ± 2.9 for AK, 0.753% ± 0.116–4.561% ± 0.630% for OC, and 0.071% ± 0.017–0.407% ± 0.048 for TN. According to the China national standard (http://www.soil17.com/news_more/1663.html, accessed on 20 August 2024), rich AN was detected in sites of Kunming and Hezhou, poor in Shihezi, and moderate to very poor in the remaining samples; AP was from moderate to very rich level in all the sites; AK was very rich in most soil samples, but moderate in Kunming; for OC, except for being very rich in Hezhou, most the soil samples were at a moderate to very poor level.

### 3.2. Peanut Rhizobial Isolation and Selection for Representative Strains

In this study, both the fast-growing and slow-growing bacteria were isolated from 3910 sampled root nodules. According to *recA* sequences, the fast-growing bacteria were mainly identified in the genera *Agrobacterium*, *Rhizobium*, *Burkholderia*, and *Sphingomonas*, and *Microbacterium*, *Dyella*, and *Clavibacter*, etc., were also observed as minor groups (data are not provided). Due to the fact that only the representative strains belonging to *Bradyrhizobium* formed root nodules on peanuts in the nodulation tests of this study, the fast-growing bacteria were excluded in the subsequent analyses. Finally, a total of 1001 *Bradyrhizobium* isolates were obtained, which presented an isolation rate of 25.60% (1001/3910) (Table S2). The isolation rates were 34.50% and 35.67% for the two fresh nodule samples and 5% (in Fenyang) to 34% (in Ganzhou) for dry nodules (Table S2). In the analysis of the *recA* sequences of all the *Bradyrhizobium* isolates, a total of 84 haplotypes were classified, and each encompassed 1–136 isolates (Table S3).

### 3.3. Phylogeny and Diversity of Peanut Rhizobia in Different Sampling Sites

**Figure 2:**
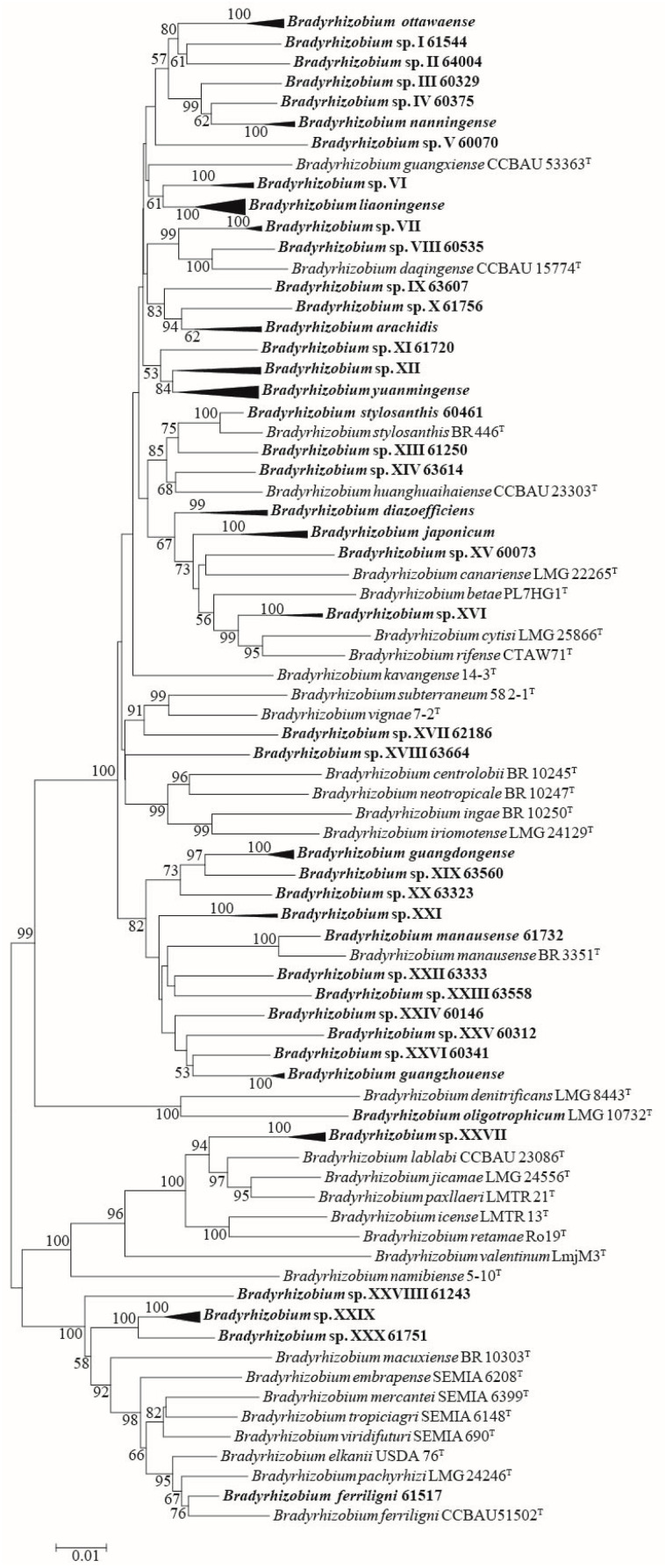
MLSA phylogenetic tree using the concatenated *dnaK* (445 nt), *glnII* (542 nt), *gyrB* (605 nt), *recA* (465 nt), and *rpoB* (771 nt) sequences. The taxa and GenBank accession numbers in bold face were obtained in this study. The tree was reconstructed using the neighbor-joining methods using MEGA 7.0, and bootstrap values greater than 50% are provided at the nodes. The scale bar represents 1% nucleotide substitution.

In MLSA, housekeeping genes *dnaK*, *glnII*, *gyrB*, and *rpoB* were also successfully amplified and sequenced for all 84 representative strains (Table S4). According to MLSA of concatenated sequences with *dnaK*, *glnII*, *gyrB*, *recA*, and *rpoB*, and compared with the sequences of related type strains (Table S5), all the *Bradyrhizobium* representative strains were divided into 42 genospecies, including 12 valid published genospecies (*B. arachidis*, *B. diazoefficiens*, *B. ferriligni*, *B. guangdongense*, *B. guangzhouense*, *B. japonicum*, *B. liaoningense*, *B. manausense*, *B. nanningense*, *B. ottawaense*, *B. stylosanthis*, and *B. yuanmingense*) and 30 undefined genospecies (Figure 2 and Table S6). The undefined genospecies accounted for 71.43% (30/42) of the total genospecies and 24.08% (241/1001 isolates) of the total isolates.

**Figure 3:**
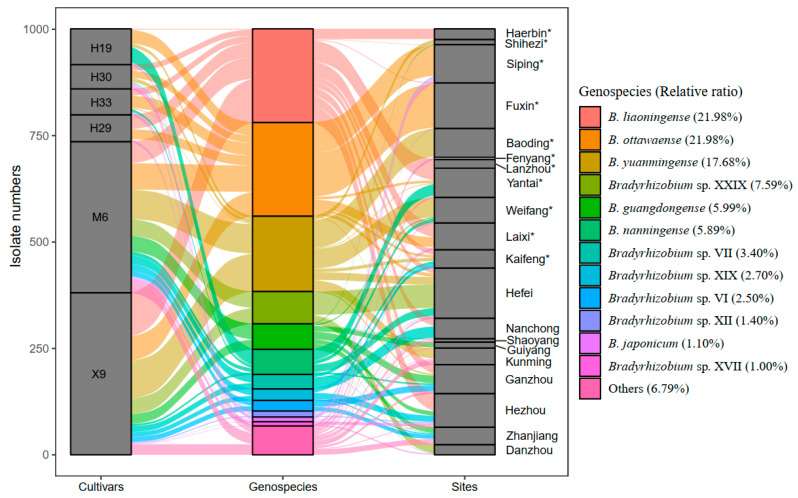
The abundance and distribution of each genospecies isolated in this study. The abscissa shows the cultivar, genospecies, and sampling sites, and the ordinate indicates the isolate number for each cultivar (left), genospecies (middle), or sampling sites (right). The height of each block indicates the isolate numbers. The sampling sites with asterisk * belong to North China, and the rest belong to South China.

Strains belonging to six genospecies were predominant (>5%), including *B. ottawaense* (220 isolates, 21.98%), *B. liaoningense* (220 isolates, 21.98%), *B. yuanmingense* (177 isolates, 17.68%), *Bradyrhizobium* sp. XXIX (76 isolates, 7.59%), *B. guangdongense* (60 isolates, 5.99%), and *B. nanningense* (59 isolates, 5.89%) (Figure 3 and Table S6). However, only a single isolate was obtained for 15 genospecies, including 2 defined species, *B. manausens* and *B. ferriligni*, and 13 undefined genospecies (Tables S3, S6 and S7). *B. liaoningense* was found to have the widest distribution, which was recorded in 12 of the 20 sampling sites, followed by *B. yuanmingense* and *B. guangdongense*, which were obtained from 8 sampling sites, respectively (Figure 3, Tables S3 and S7). Three sampling sites, Hefei, Kunming, and Zhanjiang, presented the greatest species richness (each with nine genospecies), followed by Shaoyang and Ganzhou, with eight genospecies (Figure 3, Tables S3, S6 and S7). However, only one genospecies was observed in Fenyang and Lanzhou (Figure 3, Tables S2 and S7).

The highest Shannon–Wiener index (*H*’ =2.08) was observed in Shaoyang, followed by Zhanjiang (*H*’ = 1.83) and Ganzhou (*H*’ = 1.65); the lowest value (0) was found in Lanzhou and Fenyang, for only a single genospecies isolated from both sites (Table S2). The remaining sampling sites with *H*’ values varied between 0.15 and 1.57. The Simpson index (*D*) values ranged from 0 in Fenyang and Lanzhou to 2.08 in Shaoyang, and the evenness index and Pielou index (*J*) values varied between 0.139 in Baoding and Fuxin and 1.000 in Shaoyang; furthermore, the *D* and *J* values showed a consistent tendency with *H*’ values (Table S2).

### 3.4. Correlation Between Soil, Climate Characteristics, and the Distribution of Peanut Rhizobia

**Figure 4:**
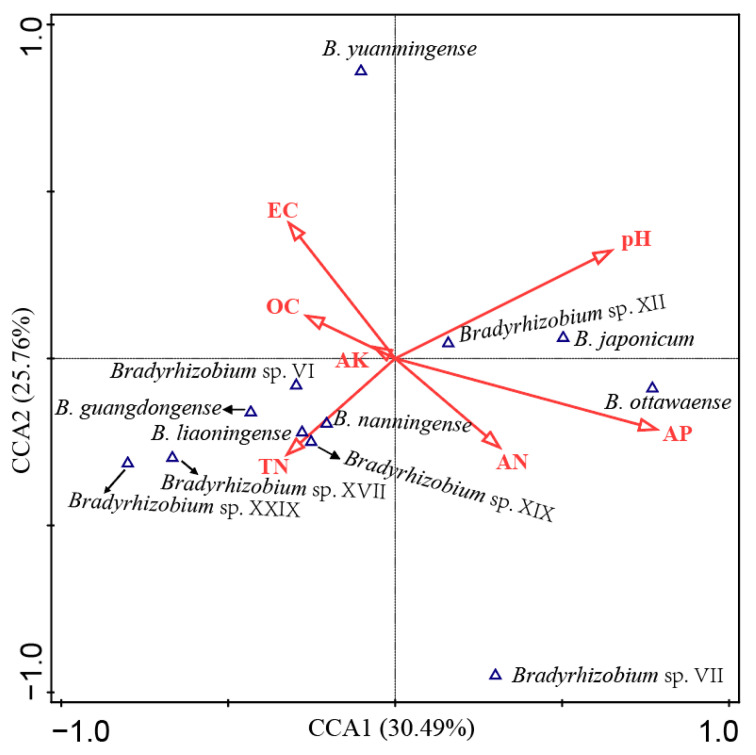
Correlation analyses between the rhizobial genospecies and soil physiochemical characteristics calculated using CANOCO 5.0. AN, available nitrogen; AP, available phosphorous; AK, available potassium; OC, organic carbon; TN, total nitrogen; EC, electrical conductivity.

A total of 12 genospecies accounting for more than 1% of total isolates were selected to perform the correlation analyses between soil characteristics and peanut rhizobia by using Canoco 5.0 (Figure 4). A length of 3.6 of the gradient (first axis) was obtained while evaluating the peanut rhizobia community through the detrended correspondence analysis (DCA) program; thus, the canonical correspondence analysis (CCA) was applied to further conduct the data analysis. The first two axes contributed 30.49% and 25.76% of the total variance explanation rate for the species distribution. This result also indicates that soil AP and pH were the main soil factors affecting the distribution of peanut rhizobia in this study, followed by EC, AN, and TN (Figure 4). The statistical analysis also demonstrated that AP and pH were factors with significant (*p* = 0.026 and 0.018, respectively) effects on the rhizobial distribution, which explains that 12.3% and 10.5% and contributed 22.5% and 19.2% of the total variation (Table S8). In detail (Figure 4), AP was positively correlated with the distribution of *B. ottawaense*, *B. japonicum*, and *Bradyrhizobium* sp. XII, while AN showed similar but minor effects, and EC presented opposite effects compared to AP. The pH positively correlated with *Bradyrhizobium* sp. XII and *B. japonicum* but negatively with *Bradyrhizobium* sp. VI, *B. guangdongense*, *B. liaoningense*, *B. nanningense*, *Bradyrhizobium* sp. XIX, *Bradyrhizobium* sp. XVII, and *Bradyrhizobium* sp. XXIX, while TN showed the opposite effects compared to pH. OC and AK showed minor correlations with the distribution of peanut rhizobial genospecies (Figure 4).

According to the CCA analyses between climate variables and the peanut rhizobium genospecies, the first two axes contributed 55.39% and 33.03% of the total variance explanation rate for the species distribution. MAT and MAP had significant effects (*p* = 0.002 and 0.04, respectively) on the peanut rhizobium genospecies distribution; they explained 13.5% and 9.4% and contributed 41.8% and 29.1% of the total variation (Table S9). Both MAP and MAT showed a similar effect on the rhizobium genospecies; they showed a positive correlation with 7 of the 12 genospecies, including *B. liaoningense*, *B. nanningense*, *Bradyrhizobium* sp. XIX, *Bradyrhizobium* sp. XXIX, *Bradyrhizobium* sp. XVII, *B. guangdongense*, and *Bradyrhizobium* sp. VI. However, PS showed reverse effects with MAP and MAT; it showed negative effects on the above genospecies. TS showed a positive correlation with *B. nanningense* and *B. ottawaense* (Figure S1). The correlation analysis results are consistent with the environmental tolerance range of each genospecies (Table S10).

### 3.5. Phylogenies of Symbiotic Genes and Nodulation Capacity of Peanut Rhizobia

Among the representative strains, five (each representing genospecies with a single isolate) failed in the amplification of symbiotic genes (Table S4), in which *Bradyrhizobium* sp. XIV 63614 and *Bradyrhizobium* sp. XVIII 63664 harbored *nifH* but without *nodC*, while *Bradyrhizobium* sp. XIII 61250, *Bradyrhizobium* sp. XXVI 60341, and *Bradyrhizobium* sp. XXVIII 61243 failed in amplifying both *nodC* and *nifH*. According to the phylogenetic relationships, all the acquired *nodC* sequences from the remaining representative strains were classified as four clusters (Figure S2). Cluster I comprises 69 representative strains and is intermingled with the type strains for nine defined *Bradyrhizobium* nodulating with *Lespedeza*, *Glycine max*, *Vigna*, and *Arachis*. Cluster II is composed of seven representative strains belonging to *B. ferriligni* and *Bradyrhizobium* sp. XXIX, which are clustered with *B. elkanii* IFO 14791^T^ (soybean) and *B. ferriligni* CCBAU 51502^T^ (*Erythrophleum*). Cluster III only comprises a single strain corresponding to *Bradyrhizobium* sp. XXX 61751 that formed an independent lineage. Cluster IV comprises four representative strains belonging to *Bradyrhizobium* sp. XXVII and is grouped with type strains of six defined species nodulating with *LabLab*, *Phaseolus lunatus*, *Retama*, and *Lupinus*.

In the *nifH* phylogenetic tree (Figure S3), the representative strains are classified into five clusters, which were consistent in general with the phylogeny of *nodC*. *nifH* clusters 1, 3, and 4 covered most of the representative strains and type strains for defined *Bradyrhizobium* species in *nodC* cluster I, as well as the *nodC* absent strains of *Bradyrhizobium* sp. XIV 63614 and sp. XVIII 63664. *nifH* cluster 2 comprises all seven representative and type strains in the *nodC* cluster II and the *nodC* cluster III strain *Bradyrhizobium* sp. XXX 61751. *nifH* cluster 5 comprises all four representative strains and six type strains in *nodC* cluster IV, as well as a representative strain in *nodC* cluster I.

The nodulation test results were consistent with the amplification of symbiotic genes (Table S4) since all the representative strains (79 strains belong to 37 genospecies) with both *nodC* and *nifH* could form effective nodules and perform effective symbiotic nitrogen fixation with peanuts. Meanwhile, the *nodC/nifH* absent strains also failed to nodulate peanut seedlings.

## 4. Discussion

### 4.1. Soil Characteristics Varied in Different Sampling Sites

Previously, several studies on the diversity and biogeography of peanut rhizobia have been reported. Although some fast-growing rhizobial strains have been isolated from the peanut nodules [11,12,13,14,51], *Bradyrhizobium* species were the main symbionts of this plant in most of the related studies, and as many as 17 defined *Bradyrhizobium* species [15,17,18,20,21,52,53] and 14 putative novel (geno)species [15,26] have been described for these rhizobia. In the present study, the isolation results were consistent with the former studies that peanuts mainly nodulated with *Bradyrhizobium* spp. across the world [4,11,15,17,21,31,53,54,55,56]. However, a main point different from the previous studies is that our present study is based upon a rhizobial collection from a vast area, covering all four climate zones across the tropical, subtropical, and temperate regions, covering nine soil types with pH ranging from very acid to moderately alkaline and with greatly varied contents of the nutrients (N, P, and K) and salinity (EC). According to the China national standard, most of the soil samples showed a shortage of OC (18/20) and AN (12/20), with the fertility level ranging from poor to very poor (Table 1). The low level of AN in the soils illustrates that peanut rhizobium plays a critical role in peanut growth and development in the sampling sites. However, all the sampling sites showed highly abundant amounts of AP and AK, especially very rich in AK, which was detected in 18 soil samples (Table 1). With the Qinling Mountains and Huaihe River as the North–South boundary of China [57], nine sites from the bottom up in Table 1 (Danzhou to Hefei) are located in South China, and the remaining 11 sampling sites are located in North China. Consistent with the previous report [58], the pH values are acid (4.92–6.10) in most soil samples from South China, except Nanchong and Guiyang (pH 7.96 and 7.12), which were obviously lower than that in North China (6.90–8.64) (*p* < 0.01).

### 4.2. Peanut Rhizobia with High Genetic Diversity in China

Corresponding to the great variations in climate and soil conditions among the sampling sites, as many as 84 *recA* haplotypes within 42 genospecies were identified among the 1001 *Bradyrhizobium* isolates in this study (Table S6). Among these genospecies, 79 *recA* haplotypes in 37 genospecies with both *nodC* and *nifH* proved to have effective symbiosis with peanuts (Table S4). Thus, although peanut–rhizobium symbiosis is established through the primitive crack entry [59], the symbiotic process is still strictly Nod factor-dependent. The detection of considerable diverse peanut *Bradyrhizobium* genospecies in this study is consistent with the observations in previous studies on peanut *Bradyrhzobium* symbionts in South America and China, etc. [14,15,60]. Both the previous and the present studies evidenced that peanut is a promiscuous host for rhizobia with diverse genetic backgrounds, such as strains belonging to *Rhizobium* and *Sinorhizobium*, as well as more than 40 *Bradyrhizobium* (geno)species. This promiscuous characteristic differed peanuts from plants that vigorously select the rhizobial genomic background, such as alfalfa, which mainly nodulates with *Sinorhizobum meliloti* or *Sinorhizobium medicae* [61]. Its promiscuous nodulation may play an important role in the wide cultivation of peanuts in the world. The six predominant genospecies (>5%) (*B. guangdongense* 6.0%, *B. liaoningense* 22.0%, *B. manausense* 5.9%, *B. nanningense* 22.0%, *B. yuanmingense* 17.7%, and *Bradyrhizobium* sp. XXIX 7.5%) observed in this study (Figure 3) were consistent with previous studies that reported that they were dominant in Shandong, Guangdong, and Henan Provinces in China and South America countries [3,15,26,62]. The predominance of the above genospecies indicated that they may have co-evolved with peanuts longer than other non-dominant species, and they could be selected with priority during the competitive nodulation process in rhizosphere soil [17].

For rhizobial species, nodulation characters are valuable traits, and the symbiosis genes may have phylogenetic relationships different from those of the genus/species affiliation in many cases, such as rhizobia associated with soybean [63], common bean [64], *Sesbania* [44], and so on. A similar situation was also detected in the present study since in the *nodC* cluster I (Figure S2), strains are in as many as 15 defined species and 20 unclassified genospecies gene sequences. These cases might imply the lateral transfer of symbiosis genes among the peanut bradyrhizobia, as suggested in rhizobia associated with other legumes [44,63,64]. Another point worthy of mentioning is the division of strains in the *nodC* cluster I into four *nifH* clusters, similar to the report by Chen et al. [15], which further evidenced the evolutionary difference between the *nod* and *nif* genes. Furthermore, the definition of diverse *nodC* and *nifH* lineages among the peanut bradyrhizobia (Figures S1 and S2) might be evidence that the peanut had no stringent selection in the symbiosis gene background for its microsymbionts.

In the present study, 36 (geno)species were identified as minor (*B. japonicum* and *Bradyrhizobium* spp. VI, VII, XII, XVII, and XIX, with frequencies 1.0–3.4%) or rare groups with a single strain to seven strains (0.1–0.7%). These minor/rare species may have no significant contribution to nitrogen fixation, but they have an important contribution to diversity. And these minor/rare species could offer the ability or possibility for peanuts to associate with the most adapted rhizobia under various conditions. Also, the finding of 30 unclassified species demonstrated that the diversity of *Bradyrhizobium* species is still underestimated. The presence of many undefined genospecies also indicates a gap in understanding both the accurate taxonomy position and genetic organization of these strains. Our following studies will further elucidate their application potential through subsequent experiments, including genomic analysis of these strains and their interactions with peanuts and other species.

A low isolation rate for peanut rhizobium was found in our study (5.00–35.67%, Table S2), consistent with previous studies on peanut rhizobium from Argentina and Brazil [3,65]. The low isolation rate may be due to the following four reasons. First, regarding the effectiveness of nodule surface sterilization, ineffective sterilization could result in the failure of isolation for the proliferation of bacteria or fungi from the nodule surface, which usually grow faster and could compete with or inhibit the slow-growing peanut rhizobium. In our study, no colony was found in the YMA plates inoculated with 100 µL of the water from the last rinse; thus, we consider the surface sterilization to be successful, and surface contamination did not occur in this study. The second reason is the presence of fast-growing, nonsymbiotic endophytic bacteria in the nodules, as observed previously [66,67] and in the present study, since they grew fast and formed competition or inhibition in the growth of *Bradyrhizobium* strains. Despite the fast-growing endophytes, the failure of amplification of *nodC* and *nifH*, as well as nodulation, in five strains (representing *recA* haplotypes/genospecies 43/XIII, 44/XIV, 54/XVIII, 68/XXVI, and 75/XXVIII) (Tables S4 and S6) also demonstrated the existence of nonsymbiotic endophytic bradyrhizobia in peanut nodules. Indeed, about less than 10% of the plate was contaminated by fast-growing bacteria or fungi in our study; they grew fast and resulted in no single colony being obtained from the plates. The third reason is the differentiation of the bacteroids of peanut bradyrhizobia in the root nodules. In peanut root nodules, terminal differentiated bacteroids with a large round swollen appearance were observed, which harbored multiploidy chromosomes and were difficult to retro-differentiate to a free living state [68]. The last reason is that peanuts usually nodulate with diverse undefined rhizobial species; the uncultured/unknown species may have specific catabolism characteristics and be difficult to cultivate using regular media such as a YMA plate. During this study, about 20% of the plate did not form any colony on the isolation plate. Our following studies could combine unculture methods, such as amplicon sequencing, metagenome analyses, and culturomic methods, to comprehensively elucidate the real reason for the low isolation rate of peanut rhizobium.

### 4.3. Peanut Rhizobia Distribute in Southern China with Higher Diversity Indices

**Figure 5:**
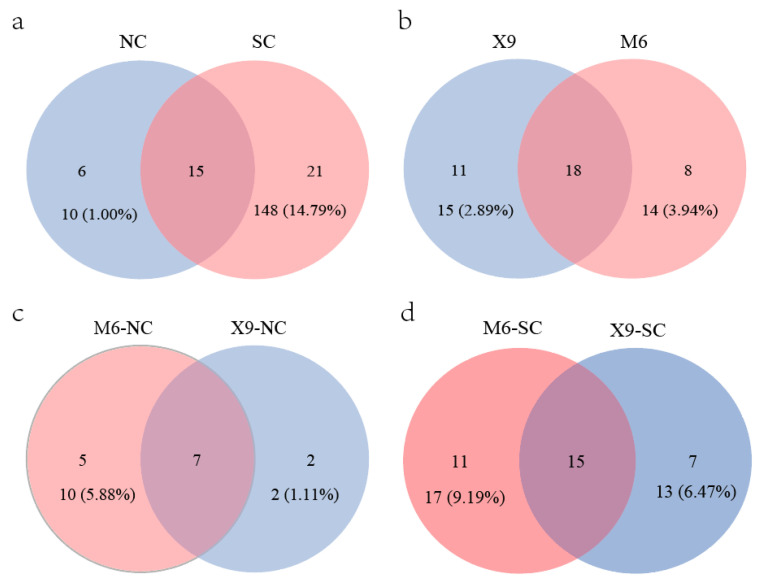
Genospecies numbers isolated from different locations or cultivars. (**a**) Genospecies distributed in North and South China; (**b**) genospecies were isolated from X9 and M6 across China; (**c**) genospecies isolated from M6 and X9 cultivated in North China; (**d**) genospecies isolated from M6 and X9 cultivated in South China. The isolate numbers and the relative abundances for the isolates from the corresponding cultivar were also provided (**b**–**d**). NC, North China, SC, South China.

Previous studies indicate that plants presented higher diversity indices in South China than in North China [69,70], which is divided by the Qinling Mountains and Huaihe River. The same phenomenon is also observed for peanut rhizobia in this study. Although more isolates were obtained from North China (562 isolates) than those from South China (439 isolates), more genospecies were found in South China (36 genospecies) than in North China (21genospecies) (Figure 5a, Table S11). A total of 15 genospecies were distributed in both parts, while 21 and 6 genospecies were specifically distributed in South and North China, respectively (Figure 5a). In line with this, the alpha diversity indices of Shannon–Wiener, Simpson, and Pielou indexes in the peanut *Bradyrhizobium* community from North China were obviously lower than in South China (Figure S3 and Table S12). The difference in peanut rhizobial community structures in South and North China might be related to the climate and soil characteristics (Table 1), and the greater rhizobial diversity in South China might be related to the longer culturing history of peanuts and the natural distribution of the wild peanut *Arachis duranensis* [71] in South China.

### 4.4. The Community of Peanut Rhizobia Was Mainly Affected by Soil Environmental Factors

Soil physiochemical characteristics are the main factors that determine the rhizobial distribution, such as pH for soybean and *Sesbania* rhizobia [24,44]. According to the correlation analyses between peanut rhizobial genospecies and soil physiochemical characteristics (Figure 4), the distribution of 9 of the 12 dominant and minor genospecies (>1%) was mainly affected by pH and TN. While *B. japonicum* and *Bradyrhizobium* sp. XII were positively affected by pH, the other seven genospecies were negatively correlated with pH and positively correlated with TN. These correlations were consistent with the fact that these seven genospecies were only or mainly distributed in the acid soils in South China (Table S8). The genospecies *Bradyrhizobium* sp. XXIX was specifically distributed in South China, consistent with it being negatively correlated with pH and positively correlated with TN (Figure 4; Table S11). And the genospecies *B. guangdongense* (55/60) was mainly distributed in South China (Table S11), consistent with its negative correlation with soil pH, which was also consistent with its dominance in acid soils of Henan and Guangdong provinces [15,26,31]. The bacterial community distributed with a large geographic scale could differ due to the effect of climate variables such as MAT and MAP [27]. MAT and MAP were also the major factors in the different peanut rhizobium genospecies distributions (Figure S1 and Table S9). Seven of the twelve genospecies selected to evaluate the correlation analyses with climate variables showed a positive correlation with MAP and MAT, which is consistent with the genospecies mainly being distributed in South China, which showed higher MAP and MAT, even with higher biodiversity [57]. And thus, higher peanut rhizobium diversity indices in South China significantly correlate with MAP, MAT, AP, and pH values.

A total of 37 *Bradyrhizobium* genospecies (736 isolates) were isolated from the two main cultivars (X9 and M6) across China, and 18 genospecies were shared by both cultivars (Figure 5b). Although 11 and 8 genospecies were specifically isolated from root nodules of X9 and M6, respectively, they only account for 2.89% (15/381 isolates) and 3.94% (14/355 isolates) of the total isolates. In North China, only 14 genospecies were isolated, among which seven were simultaneously isolated from both cultivars, two and five were specifically isolated from X9 and M6, which account for only 1.11% (2/180 isolates) and 5.88% (10/170 isolates) (Figure 5c, Table S6). While in South China, 33 species were isolated, of which 15 were common for both cultivars, with isolates accounting for 91.54% and 92.97% of X9 and M6 (Figure 5d, Table S6), respectively. Seven (13 isolates) and eleven (17 isolates) genospecies were specifically isolated from X9 and M6, which accounted for 6.47% and 9.19% of the isolates, respectively. According to the above description, the dominant/minor rhizobial genospecies were common for both cultivars across China, and specific genospecies isolated from either cultivar only comprise a small proportion. Furthermore, no obvious differences were observed for the peanut rhizobial diversity indices between the two cultivars (Figure S4), indicating that both peanut cultivars showed weak preference selection in the peanut rhizobial genospecies in our study. This was consistent with the observation that the rhizosphere bacterial community was mainly shaped by soil environment factors [72]. Above all, obvious differences in the diversity indices of peanut rhizobia were observed between North and South China but not between the main cultivars (X9 and M6). Furthermore, the MANOVA analyses for the interaction between peanut cultivars and rhizobium genospecies indicated no significant selection effect of peanut cultivars on rhizobium genospecies (*p* = 1.0) (Tables S13 and S14), which also illustrates the soil environmental factors but not peanut cultivar as the major force shaping the peanut rhizobial community in our study. It is interesting to further evaluate the peanut selection on rhizobium genospecies by comparing the rhizobium communities from more peanut genotypes/cultivars.

There are also limitations for our study due to the bacterial community (including rhizobium) in the soil being shaped by multiple both abiotic and biotic factors [73]. Apart from soil environmental factors evaluated in this study, other abiotic factors, such as fertilization practice management; tillage measure; and biotic factors, including the regulation of root exudate and the interaction between peanut rhizobium and other microorganisms in the overall microbial community of peanut rhizosphere, soil could significantly affect the peanut rhizobium genospecies distribution, competitive nodulation, and even nitrogen fixation efficiency. In addition, screening efficient strains and evaluating their survival rate in soil, as well as the application effects on a large geographic scale, could be performed in following studies.

## 5. Conclusions

Peanut bradyrhizobia isolated from 20 sites across China were classified as 42 *Bradyrhizobium* genospecies, including 12 defined and 30 undefined genospecies, and only the strains with both *nod* and *nif* genes could form an effective symbiosis with peanuts. The genospecies *B. ottawaense*, *B. liaoningense*, *B. yuanmingense*, *Bradyrhizobium* sp. XXIX, *B. guangdongense*, and *B. nanningense* were dominant groups across China, and the rhizobial community was mainly shaped by soil environmental factors (MAP, MAT, pH, and AP), and a greater diversity of peanut rhizobia was detected in South China than that in North China. Combined with the geographic distribution of peanut rhizobium and the correlational analyses with the environmental factors, our study illustrates that before the application of peanut rhizobium inoculants, it is necessary to consider the soil environmental adaptability and competitive nodulation ability of the relevant strains.

## Figures and Tables

**Table 1:** Properties of soil samples and diversity analyses for each sampling site.

Sampling Sites	AK (mg/kg)	AP (mg/kg)	AN (mg/kg)	TN%	OC%	EC (μs/cm)	pH	Fertility Level ^A^ (OC/AN/AP/AK)	MAP (mL)	MAT (°C)	PS	TS
Haerbin	437.0 ± 2.9 ^a^	41.1 ± 3.2 ^b,c^	94.5 ± 1.8 ^c,d^	0.214 ± 0.012 ^b,c^	1.462 ± 0.521 ^f,g^	170.0 ± 6.8 ^d,e^	6.97 ± 0.16 ^e^	4/3/1/1	530	2.2	1.10	6.83
Siping	363.8 ± 5.3 ^d^	37.5 ± 1.8 ^b,c^	102.9 ± 2.3 ^c^	0.122 ± 0.007 ^c,d,e^	1.178 ± 0.362 ^h,i^	52.8 ± 4.5 ^j^	8.22 ± 0.37 ^a,b^	4/3/2/1	618	8.1	1.04	1.48
Fuxin	239.5 ± 3.2 ^i^	82.8 ± 1.3 ^a^	91.6 ± 0.8 ^d^	0.121 ± 0.041 ^c,d,e^	1.145 ± 0.008 ^h,i^	69.4 ± 3.6 ^i^	7.32 ± 0.33 ^d^	4/3/1/1	503	7.8	1.10	1.57
Shihezi	317.4 ± 8.6 ^e^	14.5 ± 0.9 ^f,g^	26.8 ± 3.6 ^h^	0.071 ± 0.017 ^e^	1.230 ± 0.517 ^g,h^	164.5 ± 10.3 ^e^	8.13 ± 0.26 ^b^	4/6/3/1	153	8.3	0.51	1.70
Baoding	251.1 ± 3.2 ^h^	22.4 ± 2.3 ^e^	37.1 ± 1.4 ^g^	0.123 ± 0.076 ^c,d,e^	2.910 ± 0.006 ^b^	131.3 ± 6.1 ^f^	8.11 ± 0.08 ^b^	3/5/2/1	507	13.2	1.29	0.80
Fenyang	326.2 ± 7.6 ^e^	11.3 ± 1.4 ^h^	80.5 ± 2.1 ^e^	0.082 ± 0.023 ^e^	2.290 ± 0.651 ^d^	208.0 ± 9.6 ^b^	8.64 ± 0.20 ^a^	3/4/3/1	462	10.4	1.01	0.96
Lanzhou	269.1 ± 2.3 ^g^	11.0 ± 0.8 ^h^	81.2 ± 3.3 ^e^	0.112 ± 0.034 ^c,d,e^	0.881 ± 0.23 ^j,k^	288.0 ± 4.3 ^a^	6.90 ± 0.08 ^e^	5/4/3/1	345	8.7	0.89	1.09
Weifang	395.6 ± 4.3 ^c^	32.0 ± 2.3 ^c^	73.1 ± 1.8 ^e^	0.104 ± 0.019 ^d,e^	1.089 ± 0.108 ^h,i,j^	172.9 ± 2.8 ^d^	7.99 ± 0.17 ^b^	4/4/2/1	690	13.0	1.06	0.77
Laixi	291.8 ± 1.9 ^f^	32.9 ± 0.7 ^c^	58.3 ± 3.5 ^f^	0.080 ± 0.010 ^e^	0.754 ± 0.093 ^k^	139.4 ± 7.4 ^f^	6.95 ± 0.04 ^e^	5/5/2/1	698	12.8	0.92	0.73
Yantai	237.2 ± 2.8 ^i^	20.6 ± 3.2 ^e,f^	38.5 ± 4.3 ^g^	0.079 ± 0.021 ^e^	0.753 ± 0.116 ^k^	104.0 ± 6.5 ^g^	7.66 ± 0.29 ^c,d^	5/5/2/1	698	12.0	0.93	0.78
Kaifeng	314.0 ± 3.2 ^e^	42.5 ± 2.8 ^b,c^	61.8 ± 1.8 ^f^	0.104 ± 0.028 ^d,e^	0.934 ± 0.237 ^i,j,k^	160.7 ± 3.3 ^e^	8.31 ± 0.13 ^a,b^	5/4/1/1	628	14.6	0.87	0.65
Hefei	315.1 ± 6.6 ^e^	27.2 ± 3.4 ^d,e^	38.3 ± 2.8 ^g^	0.139 ± 0.043 ^c,d,e^	1.041 ± 0.108 ^h,i,j^	92.1 ± 4.8 ^h^	5.85 ± 0.21 ^f,g^	4/5/2/1	1146	16.0	0.52	0.55
Ganzhou	199.4 ± 2.7 ^j^	41.1 ± 1.5 ^bc^	119.0 ± 5.6 ^b^	0.134 ± 0.012 ^c,d,e^	1.043 ± 0.072 ^h,i,j^	70.6 ± 7.7 ^i^	5.81 ± 0.32 ^f,g^	4/3/1/2	1492	18.9	0.50	0.38
Shaoyang	294.4 ± 3.6 ^f^	46.3 ± 2.5 ^b^	71.8 ± 6.4 ^e^	0.180 ± 0.046 ^b,c,d^	1.002 ± 0.009 ^i,j,k^	134.1 ± 2.7 ^f^	4.92 ± 0.23 ^h^	5/4/1/1	1353	17.3	0.47	0.46
Nanchong	228.6 ± 4.3 ^i^	29.7 ± 0.8 ^d^	98.7 ± 3.6 ^c,d^	0.153 ± 0.008 ^c,d,e^	1.101 ± 0.211 ^h,i,j^	106.2 ± 5.5 ^g^	7.96 ± 0.09 ^b,c^	4/3/2/1	1090	17.5	0.78	0.41
Guiyang	264.4 ± 5.9 ^g^	10.1 ± 1.6 ^h^	94.9 ± 1.7 ^c,d^	0.252 ± 0.025 ^b^	2.603 ± 0.139 ^c^	198.0 ± 8.6 ^c^	7.12 ± 0.17 ^e^	3/3/3/1	1113	15.2	0.68	0.43
Kunming	199.2 ± 5.1 ^j^	13.3 ± 2.3 ^h^	141.2 ± 2.5 ^a^	0.117 ± 0.036 ^c,d,e^	0.844 ± 0.097 ^j,k^	169.5 ± 8.9 ^d,e^	6.10 ± 0.35 ^f^	5/2/3/2	928	14.9	0.81	0.29
Hezhou	412.2 ± 6.8 ^b^	34.2 ± 2.1 ^c^	122.2 ± 6.8 ^b^	0.407 ± 0.048 ^a^	4.561 ± 0.630 ^a^	70.2 ± 1.2 ^i^	5.68 ± 0.17 ^g^	1/2/2/1	1567	20.1	0.59	0.33
Zhanjiang	239.8 ± 3.8 ^i^	14.0 ± 1.8 ^h^	63.0 ± 7.3 ^f^	0.166 ± 0.053 ^b,c,d^	1.823 ± 0.026 ^e^	102.1 ± 1.5 ^g^	5.89 ± 0.23 ^f,g^	4/4/3/1	1763	23.3	0.69	0.21
Danzhou	259.1 ± 2.7 ^g,h^	12.9 ± 2.6 ^h^	73.2 ± 1.9 ^e^	0.183 ± 0.009 ^b,c,d^	1.671 ± 0.139 ^e,f^	72.1 ± 4.3 ^i^	4.92 ± 0.18 ^h^	4/4/3/1	1708	24.1	0.74	0.15

The soil’s physiochemical characteristics were analyzed in triplicate; each data piece consists of a mean and a standard deviation, and the lowercase superscript letters (a–k) indicate significant differences among different sampling sites evaluated using one-way ANOVA, Tukey’s test. ^A^ According to the National Norma of China, level 1 is very rich, 2 is rich, 3 is moderate, 4 is poor, 5 is very poor, and 6 is extremely poor (http://www.soil17.com/news_more/1663.html, accessed on 20 August 2024).

## Data Availability

All the gene sequences obtained in this study were submitted to GenBank, and the accession numbers are listed in Table S4.

## Supplementary Materials

The following supporting information can be downloaded at: https://www.mdpi.com/article/10.3390/microorganisms13040926/s1, Figure S1: Correlation analyses between the rhizobial genospecies and environmental variables, calculated using CANOCO 5.0. Figure S2: A phylogenetic tree of *nodC* sequences of representative and reference rhizobial strains. The tree was reconstructed using the neighbor-joining method. Bootstrap values greater than 50% are shown at the nodes. The scale bar represents 5% nucleotide substitutions. Figure S3: A phylogenetic tree of *nifH* sequences of representative and reference rhizobial strains. The tree was reconstructed using the neighbor-joining method. Bootstrap values greater than 50% are shown at the nodes. The scale bar represents 2% nucleotide substitutions. Figure S4: Diversity indices of peanut rhizobial community isolated from different locations or cultivars. A, B, and C represent peanut rhizobia isolated from different locations in Northern and Southern China; D, E, and F represent peanut rhizobia isolated from different cultivars of X9 and M6. a, b in the plot indicate the significance of the difference. Table S1: The information on the sampling sites and sampling date. Table S2: The isolation rate of *Bradyrhizobium* strains from each sampling site. Table S3: The distribution of *Bradyrhizobium* isolates in different sampling sites and rhizobia haplotype classification. Table S4: A detailed list of accession numbers of housekeeping genes and symbiotic gene sequences obtained in this study. Table S5: A detailed list of accession numbers of housekeeping genes of reference type strains downloaded from GenBank. Table S6: MLSA similarities of representative strains with each genospecies. Table S7: The geographic distribution of peanut rhizobium genospecies. Table S8: The detailed Canoco results of soil characteristics and genospecies. Table S9: The detailed Canoco results of climate variables and peanut rhizobium genospecies. Table S10: The tolerance range of soil environmental factors for each peanut rhizobium genospecies. Table S11: The statistics of the isolates distributed in different sites. Table S12: Alpha diversity indices for peanut rhizobium distributed in different regions or cultivars. Table S13: A multivariate test of MANOVA indicating the effect of environmental variables on peanut rhizobia obtained from different sampling sites and different peanut cultivars. Table S14: Tests of between-subjects effects of MANOVA results indicating the effect of environmental variables on peanut rhizobia obtained from different sampling sites and peanut cultivars.

## Abbreviations

X9	Peanut cultivar Xuhua 9.
M6	Peanut cultivar Minhua 6.
H19	Peanut cultivar Huayu 19.
H30	Peanut cultivar Huayu 30.
H33	Peanut cultivar Huayu 33.
H29	Peanut cultivar Huayu 29.
YMA	Yeast mannitol agar medium.
AN	Available nitrogen.
AP	Available phosphorous.
AK	Available potassium.
TN	Total nitrogen.
OC	Organic carbon.
EC	Electrical conductivity.
MAP	Mean annual precipitation.
MAT	Mean annual temperature.
PS	Precipitation seasonality.
MLSA	Multilocus sequence analysis.
