# Supplementary material for: The Effect of Climate Variables, Soil Characteristics, and Peanut Cultivars on the Rhizobial Bacteria Community
Source: Microorganisms. 2025 Apr 17;13(4):926. doi: 10.3390/microorganisms13040926 (PMC12029271; doi:10.3390/microorganisms13040926)
Supplement: Supplementary file 1 [file microorganisms-13-00926-s001.zip › microorganisms-3523856-supplementary.pdf]

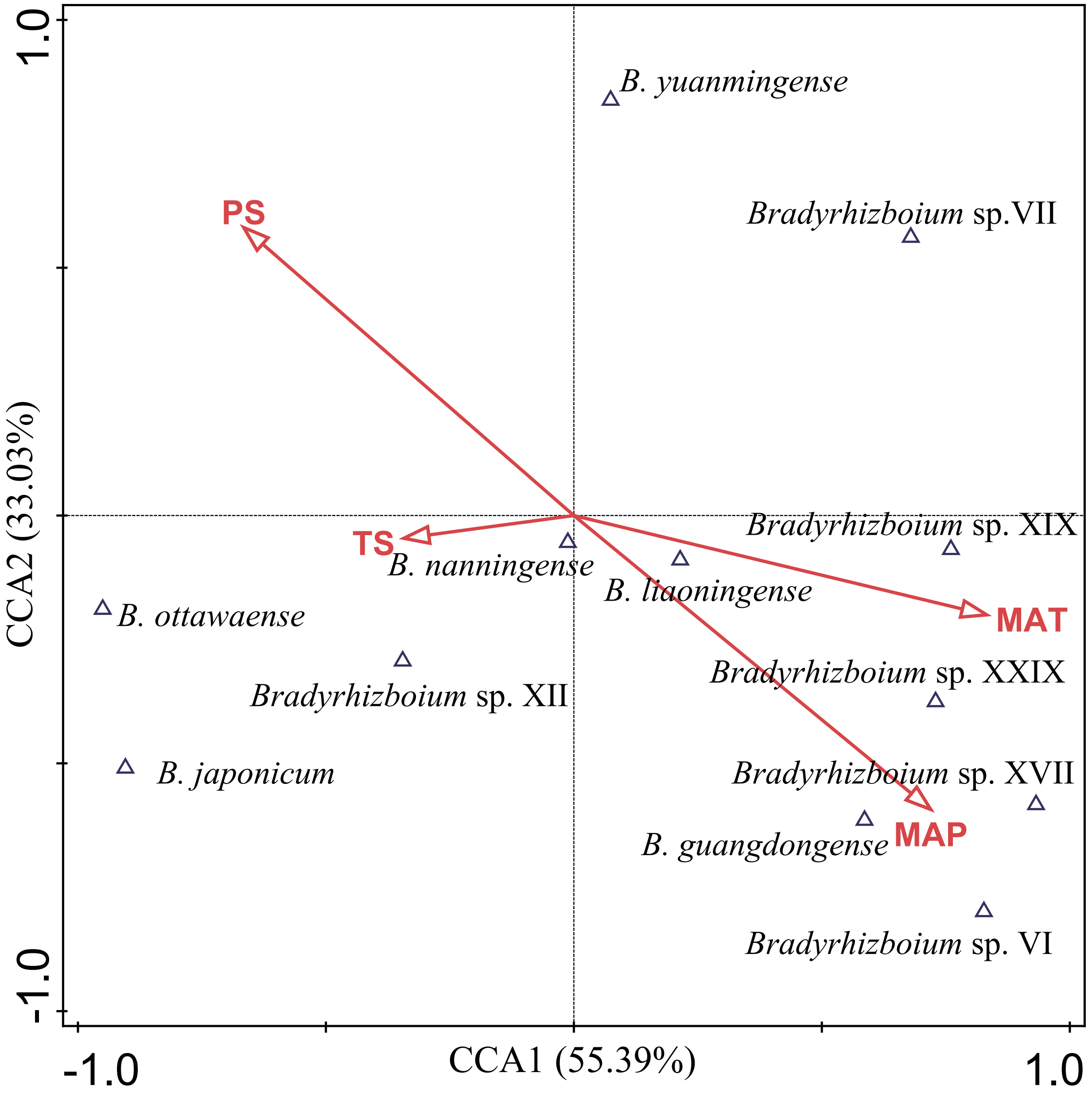

Supplementary Figure S1 Correlation analyses between the rhizobial genospecies and environmental variables, calculated using CANOCO 5.0.

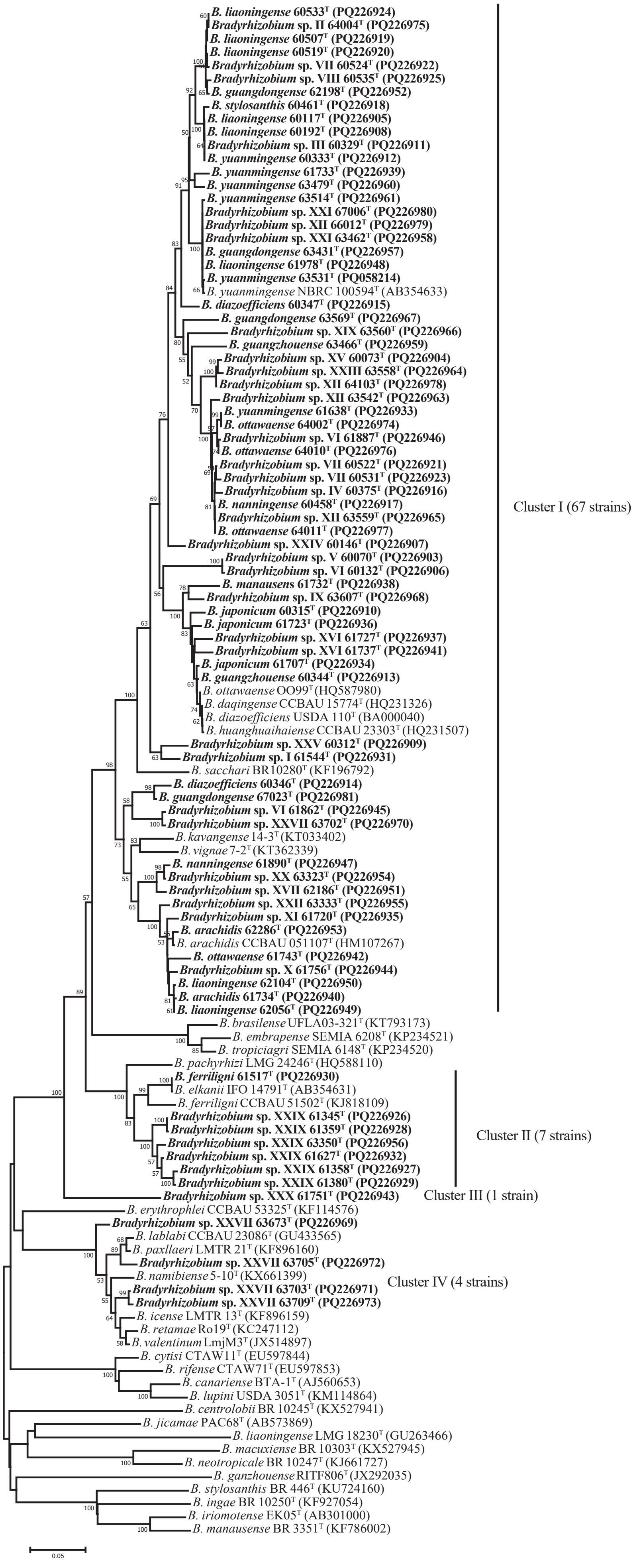

Supplementary Figure S2 A phylogenetic tree of *nodC* sequences of representative and reference rhizobial strains. The tree was reconstructed using the neighbor-joining method. Bootstrap values greater than 50% are shown at the nodes. The scale bar represents 5% nucleotide substitutions.

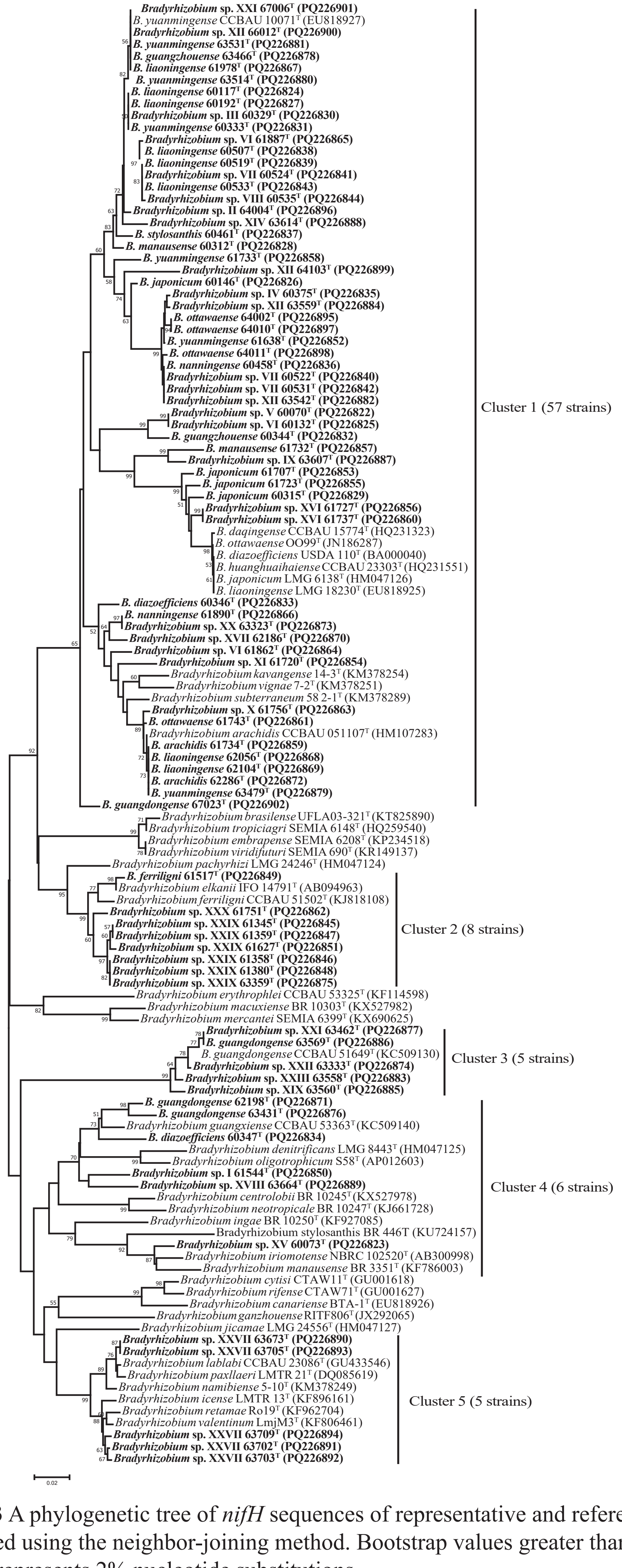

Supplementary Figure S3 A phylogenetic tree of *nifH* sequences of representative and reference rhizobial strains. The tree was reconstructed using the neighbor-joining method. Bootstrap values greater than 50% are shown at the nodes. The scale bar represents 2% nucleotide substitutions.

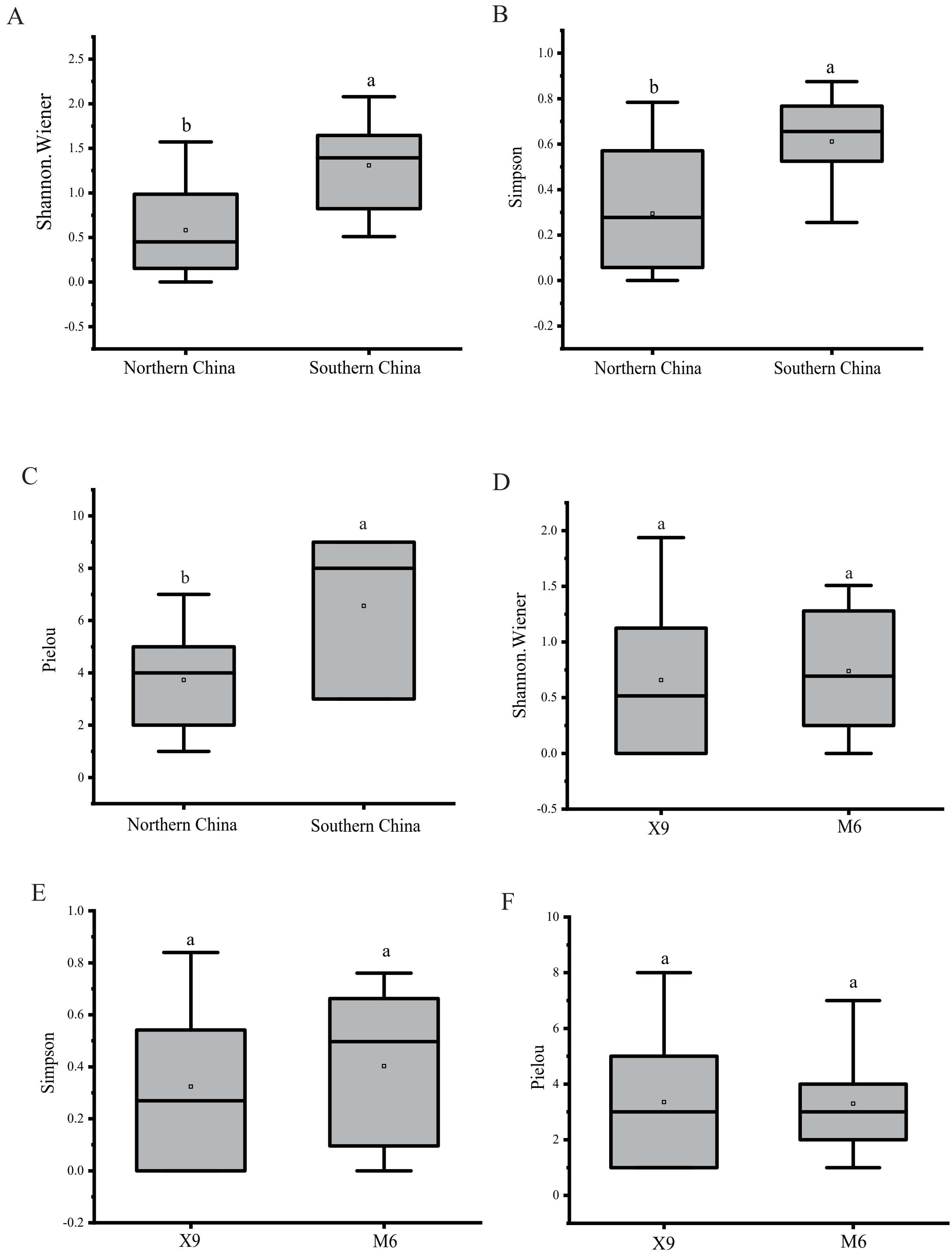

Supplementary Figure S4 Diversity indices of peanut rhizobial community isolated from different locations or cultivars. A, B, and C represent peanut rhizobia isolated from different locations in Northern and Southern China; D, E, and F represent peanut rhizobia isolated from different cultivars of X9 and M6. a, b in the plot indicate the significance of the difference.

Table S1 The information on the sampling sites and sampling date.

Sampling sites	Location	Climatic zone	Soil type	Sampling date
Haerbin	N47.46, E126.94	Temperate monsoon	Alisols	2017.07
Siping	N43.17, E124.35	Temperate monsoon	Alisols	2018.07
Fuxin	N42.02, E121.67	Temperate monsoon	Luvisols	2017.08
Shihezi	N44.32, E86.00	Temperate continental	Cambisols	2017.08
Baoding	N38.87, E115.47	Temperate monsoon	Arenosols	2018.07
Fenyang	N37.27, E111.78	Temperate continental	Luvisols	2018.07
Lanzhou	N36.12, E103.74	Temperate continental	Cambisols	2017.07
Weifang	N36.70, E119.15	Temperate monsoon	Luvisols	2017.08
Laixi	N36.34, E120.40	Temperate monsoon	Alisols	2015.07
Yantai	N37.44, E121.45	Temperate monsoon	Arenosols	2016.08
Kaifeng	N34.80, E114.30	Temperate monsoon	Cambisols	2018.08
Hefei	N31.83, E117.25	Subtropical monsoon	Alisols	2017.07, 2018.07
Ganzhou	N25.83, E114.93	Subtropical monsoon	Ferralsols	2016.08
Shaoyang	N27.25, E111.47	Subtropical monsoon	Leptosols	2017.06
Nanchong	N30.78, E106.08	Subtropical monsoon	Haplustepts	2016.08
Guiyang	N26.53, E106.70	Subtropical monsoon	Leptosols	2016.08
Kunming	N25.14, E102.75	Subtropical monsoon	Arenosols	2018.07
Hezhou	N24.42, E111.55	Subtropical monsoon	Acrudox	2018.07
Zhanjiang	N21.27, E110.35	Subtropical monsoon	Haplustepts	2018.07
Danzhou	N19.52, E109.51	Tropical monsoon	Alisols	2016.07

Table S2 The isolation rate of *Bradyrhizobium* strains from each sampling site.

Sampling sites	Isolated nodule number	Nodule type	Isolate number of *Bradyrhizobium*	Isolation rate	Species number	*H′* [a]	*D* [b]	*J* [c]
Haerbin	100	Dry	25	25.00%	2	0.17	0.08	0.242
Siping	300	Dry	90	30.00%	7	0.79	0.35	0.405
Fuxin	300	Fresh	107	35.67%	4	0.20	0.07	0.143
Shihezi	100	Dry	12	12.00%	2	0.45	0.28	0.650
Baoding	300	Dry	68	22.67%	3	0.15	0.06	0.139
Fenyang	100	Dry	5	5.00%	1	0.00	0.00	NaN
Lanzhou	100	Dry	20	20.00%	1	0.00	0.00	NaN
Weifang	300	Dry	60	20.00%	5	0.77	0.39	0.481
Laixi	200	Dry	63	31.50%	4	0.99	0.57	0.710
Yantai	200	Fresh	69	34.50%	7	1.31	0.66	0.670
Kaifeng	200	Dry	43	21.50%	5	1.57	0.78	0.976
Hefei	400	Dry	118	29.50%	9	1.57	0.72	0.716
Ganzhou	200	Dry	68	34.00%	8	1.65	0.77	0.791
Shaoyang	100	Dry	8	8.00%	8	2.08	0.88	1.000
Nanchong	200	Dry	48	24.00%	3	0.82	0.53	0.749
Guiyang	100	Dry	14	14.00%	3	0.51	0.26	0.463
Kunming	110	Dry	39	35.45%	9	1.39	0.61	0.634
Hezhou	300	Dry	79	26.33%	7	1.36	0.66	0.700
Zhanjiang	200	Dry	41	20.50%	9	1.83	0.80	0.835
Danzhou	100	Dry	24	24.00%	3	0.54	0.28	0.495
Total	3910	--	1001	25.60%	42	--	--	--

[a] *H′*, Shannon-Wiener index; [b] *D*, Simpson index; [c] *J*, Pielou index.

Table S3 The distribution of *Bradyrhizobium* isolates in different sampling sites and rhizobia haplotype classification.

recA haplotype	Representative strain	Isolates (Number)	Cultivar	Sampling stie
1	64011	60015, 60016, 60019, 60021, 60033, 60063, 60105, 60197 (8)	H19	Siping
		60118 (1)	H30	Siping
		60031, 60032, 60072, 60092, 60093, 60098, 60103, 60109, 60143, 60206, 60208 (11)	H33	Siping
		64060, 64061, 64062, 64063, 64064, 64065, 64066, 64067, 64068, 64069, 64070, 64071, 64073, 64074, 64076, 64077, 64078, 64079, 64080, 64081, 64082, 64083, 64084, 64087, 64088, 64090, 64091, 64092, 64093, 64094, 64095, 64096, 64097, 64098, 64099, 64102, 64105, 64107, 64108, 64113 (40)	X9	Fuxin
		61744, 64001, 64003, 64007, 64008, 64009, 64110, 64011, 64013, 64014, 64106, 64017, 64018, 64019, 64022, 64023, 64025, 64026, 64027, 64028, 64030, 64031, 64032, 64035, 64036, 64040, 64042, 64043, 64044, 64045, 64047, 64048, 64049, 64051, 64053, 64056, 64059, 64104, 64111, 64112 (40)	M6	Fuxin
		61549, 61550, 61552, 61553, 61567, 61575 (6)	H19	Yantai
		60813, 60840, 60841, 60894, 60896, 60897, 60898, 60907, 60961, 60910, 62250, 62252, 62271, 62280, 62283, 62284, 62287, 62291, 62296, 62302, 62307, 62311, 62323 (23)	H29	Laixi
		61287, 61299, 61302, 61303 (4)	X9	Kaifeng
		61316, 61334 (2)	M6	Kaifeng
		61607 (1)	X9	Hefei
2	61743	60008, 60009, 60010, 60012, 60013, 60014, 60017, 60020, 60024, 60034, 60058, 60059, 60060, 60062, 60064, 60196, 60210, 60245 (18)	H19	Siping

		60007, 60037, 60039, 60040, 60047, 60048, 60049, 60052, 60053, 60055, 60065, 60066, 60067, 60104, 60198, 60204 (16)	H30	Siping
		60026, 60027, 60028, 60029, 60056, 60057, 60088, 60091, 60094, 60099, 60100, 60102, 60108, 60110, 60124, 60199, 60209, 60226 (18)	H33	Siping
		64024, 64075, 64085, 64089 (4)	X9	Fuxin
		64005, 64015, 64034, 64037, 64041, 64050, 64054 (7)	M6	Fuxin
		61618, 61588, 61596, 61599, 61601, 61622 (6)	X9	Hefei
		61623 (1)	M6	Hefei
		61701 (1)	X9	Kunming
		61743 (1)	M6	Kunming
3	64002	64002, 64006, 64012, 64109, 64020, 64021, 64033, 64038, 64039, 64055 (10)	M6	Fuxin
4	64010	64058 (1)	X9	Fuxin
		64010 (1)	M6	Fuxin
5	61544	61544 (1)	H30	Yantai
6	64004	64004 (1)	M6	Fuxin
		61536, 61543, 61546 (3)	H30	Yantai
		60087 (1)	X9	Ganzhou
7	60329	60329 (1)	X9	Weifang
		60382 (1)	H19	Hefei
8	60375	60375 (1)	H30	Hefei
9	60458	60458 (1)	H19	Hefei
10	61890	61469, 61472, 61474, 61476, 61477, 61478, 61479, 61482, 61483, 61486, 61488, 61490, 61491, 61492, 61493, 61494, 61495, 61499, 61500, 61501, 61504, 61506, 61507, 61511, 61512, 61513, 61514 (27)	H19	Yantai
		60271, 60304, 60321 (3)	X9	Weifang
		60279, 60288, 60290, 60298, 60326, 60342 (6)	M6	Weifang
		60281, 60282, 60284, 60369, 60386, 60387, 60388, 60398, 60709 (9)	H19	Hefei
		60357, 60372 (2)	H30	Hefei

		60339, 60340, 60360, 60361, 60377, 60454 (6)	H33	Hefei
		61890 (1)	M6	Zhanjiang
		60035, 60097 (2)	X9	Ganzhou
		60082, 60115 (2)	M6	Ganzhou
11	60070	60070 (1)	X9	Ganzhou
12	60132	60130, 60127, 60131, 60139, 60150, 60157, 60224, 60261 (8)	X9	Ganzhou
		60126, 60132, 60133, 60133-2, 60152, 60153, 60156 (7)	M6	Ganzhou
13	61887	61851, 61870, 61880, 61887 (4)	M6	Zhanjiang
14	61862	61811, 61812, 61815, 61819 (4)	X9	Zhanjiang
		61858, 61862 (2)	M6	Zhanjiang
15	60507	60507 (1)	M6	Nanchong
16	60533	60511, 60533, 60539, 60541, 60556 (5)	X9	Nanchong
		60513, 60516, 60518, 60540, 60543, 60548 (6)	M6	Nanchong
17	60519	60519, 60565, 60581 (3)	M6	Nanchong
18	60192	60532, 60542 (2)	X9	Nanchong
		60192 (1)	X9	Shaoyang
		60004, 60086 (2)	X9	Ganzhou
		60076, 60081 (2)	M6	Ganzhou
		63291, 63298, 63300, 63311, 63326, 63329, 63352, 63356, 63557, 63587, 63596, 63597, 63601, 63603, 63604, 63619, 63621, 63628, 63651, 63653, 63654, 63655, 63656, 63657, 63658, 63659, 63661, 63662, 63668, 63669, 63670, 63671, 63672, 63674, 63690 (35)	X9	Hezhou
		62167, 62174, 62178, 62180, 62182, 62190, 62192 (7)	M6	Hezhou
19	61978	61978 (1)	X9	Haerbin
		60552, 60558 (2)	M6	Nanchong
20	60117	60044 (1)	H30	Siping
		61998, 62007, 62010, 62013, 62018, 62019, 62021, 62023, 62030, 62033, 62042 (11)	X9	Lanzhou
		62053, 62068, 62072, 62073, 62093, 62094 (6)	M6	Lanzhou
		61309, 61310, 61311-2 (3)	X9	Kaifeng

		61328, 61390 (2)	M6	Kaifeng
		64086 (1)	X9	Fuxin
		64052 (1)	M6	Fuxin
		61672, 61688, 61689 (3)	M6	Hefei
		60071, 60112, 60113, 60119, 60128, 60137, 60165 (7)	X9	Ganzhou
		60080, 60117 (2)	M6	Ganzhou
21	62056	62020，62029 (2)	X9	Lanzhou
		62056 (1)	M6	Lanzhou
		61286, 61288, 61294, 61298 (4)	X9	Kaifeng
		61318, 61321 (2)	M6	Kaifeng
		61587, 61589, 61593, 61595, 61612, 61615 (6)	X9	Hefei
		61671, 61663 (2)	M6	Hefei
22	62104	61972, 61973, 61974, 61975, 61976, 61980, 61981, 61982, 61984, 61985, 61986, 61992, 61994, 61996 (14)	X9	Haerbin
		62097, 62099, 62102, 62104, 62107, 62108 (6)	M6	Haerbin
		61547, 61548, 61551, 61555, 61557, 61559, 61570, 61571, 61572, 61574, 61577, 61582, 61584 (13)	H30	Yantai
		61518, 61519, 61520, 61521, 61522, 61523, 61524, 61525, 61526, 61528, 61532, 61535, 61537, 61538, 61515, 61516 (16)	H33	Yantai
		60746, 60747, 60748, 60749, 60752, 60756, 60765, 60781, 60784, 60785, 60792, 60838, 60890, 60891, 60893, 60902, 60903, 60908, 60911, 60912, 60927, 60935, 60936, 60944, 60945, 60947, 60949, 60962, 60963, 60964, 60967, 60968, 60969, 62318 (34)	H29	Laixi
		60247 (1)	X9	Kaifeng
		61792 (1)	M6	Kaifeng
		60332, 60378 (2)	H19	Hefei
		60114, 60120, 60147, 60154, 60155, 60189, 60200, 60257 (8)	X9	Ganzhou
		60182 (1)	M6	Ganzhou
23	60522	60522 (1)	X9	Nanchong
		60512, 60517, 60550 (3)	M6	Nanchong
24	60524	60179 (1)	X9	Kaifeng

		60172, 60173, 60177, 60184, 60193, 60237 (6)	M6	Kaifeng
		60523, 60524, 60534, 60536, 60510 (5)	X9	Nanchong
		60514, 60520, 60521, 60544, 60545, 60546, 60547, 60549, 60551, 60559, 60560 (11)	M6	Nanchong
25	60531	60508, 60509, 60531, 60537, 60538, 60555, 60557 (7)	X9	Nanchong
26	60535	60530, 60535 (2)	X9	Nanchong
27	63607	60294 (1)	X9	Shaoyang
		63607 (1)	X9	Hezhou
28	61756	61756 (1)	M6	Kunming
29	61734	61734 (1)	M6	Kunming
30	62286	62286, 62299, 62303, 62305 (4)	H29	Laixi
31	61720	60062 (1)	H19	Siping
		60006 (1)	H30	Siping
		61729 (1)	X9	Kunming
32	63542	63542, 63595 (2)	X9	Hezhou
33	63559	61510 (1)	H29	Laixi
		60334 (1)	H30	Herfei
		60460 (1)	H33	Herfei
		63559 (1)	X9	Hezhou
34	64103	60011, 60018 (2)	H19	Siping
		60001, 60069, 60121 (3)	H33	Siping
		64103 (1)	M6	Fuxin
		60205 (1)	H29	Laixi
35	66012	66012 (1)	M6	Baoding
36	60333	61473, 61496 (2)	H19	Yantai
		60333, 60350, 60368, 60370, 60383, 60453 (6)	H19	Hefei
		60353, 60358, 60364, 60366, 60376, 60381, 60456, 60457, 60462 (9)	H30	Hefei
		60337, 60356 (2)	H33	Hefei
37	63531	63765 (1)	M6	Baoding
		62511, 63461, 63495, 63508, 63510, 63512, 63518, 63519, 63520, 63521, 63530, 63531, 63532, 63535, 63590, 63676 (16)	X9	Weifang
		60269, 63473, 63475, 63482, 63483, 63484, 63486, 63487, 63489, 63491,	M6	Weifang

		63494, 63509, 63528, 63529, 63523, 63571, 63573 (17)		
		60258 (1)	X9	Kaifeng
		60159, 60162, 60174, 60292 (4)	X9	Ganzhou
		61762, 61776, 61779, 61781, 61782, 61783, 61785, 61786, 61787 (9)	X9	Shihezi
38	61733	61702 (1)	X9	Kunming
		61733 (1)	M6	Kunming
		63478, 63479 (2)	M6	Weifang
39	63479	60253, 60255, 60266 (3)	X9	Kaifeng
		60160, 60169, 60252 (3)	M6	Kaifeng
40	61638	61638 (1)	X9	Shihezi
		62129, 62130, 62131, 62132, 62133, 62134, 62135, 62136, 62137, 62138, 62140, 62141, 62142, 62143, 62144, 62146, 62148, 62149, 62150, 62151, 62152, 62153, 62155, 62156, 62158, 62159, 62160, 62330, 63663, 63677, 63680, 63682, 63683, 63684, 63686, 63688, 63691, 63698 (38)	X9	Baoding
		62145, 62210, 62212, 62213, 62215, 62216, 62221, 62217, 62222, 62223, 62224, 62225, 62227, 62230, 62232, 62233, 62238, 62239, 62240, 62241, 62242, 62243, 62245, 62246, 62247, 63763, 63764 (27)	M6	Baoding
41	63514	60203, 60264, 60277, 60305, 63514, 63517 (6)	X9	Weifang
		60325, 63626, 63505, 63570, 63593 (5)	M6	Weifang
		61338 (1)	X9	Kaifeng
		61656 (1)	X9	Hefei
		61698，61700，61703，61704, 61705, 61708, 61709, 61715, 61719 (9)	X9	Kunming
		61745, 61747, 61748，61750, 61754, 61755, 61758, 61759-1, 61759-2, 61722, 61728, 61736 (12)	M6	Kunming
42	60461	60379, 60461 (2)	H33	Hefei
43	61250	61250 (1)	M6	Guiyang
44	63614	63614 (1)	M6	Weifang
45	60346	60346 (1)	M6	Shaoyang

46	60347	60347 (1)	H33	Siping
47	60315	60315 (1)	M6	Shaoyang
48	61707	61707 (1)	X9	Kunming
49	61723	60038, 60043, 60050, 60090, 60106, 60123, 60202, 60207 (8)	H30	Siping
		61723 (1)	M6	Kunming
50	60073	60073 (1)	H19	Siping
51	61727	61727, 61729, 61730, 61739, 61753, 61757 (6)	M6	Kunming
52	61737	61737 (1)	M6	Kunming
53	62186	63328 (1)	X9	Hezhou
		62179, 62186, 62193, 62200, 62201, 62207 (6)	M6	Hezhou
		61382, 61386, 61399 (3)	M6	Danzhou
54	63664	63664 (1)	X9	Baoding
55	62198	60136 (1)	X9	Ganzhou
		60078，60079, 60111, 60135, 60140, 60141-2, 60201, 60211, 60213 (9)	M6	Ganzhou
		61204, 61205, 62206, 62164, 62168, 62169, 62171, 62175, 62177, 62181, 62195, 62198 (12)	X9	Guiyang
		63321, 63327, 63584, 63586, 63599, 63602, 63606 (7)	M6	Hezhou
		61388 (1)	M6	Danzhou
56	63431	61632, 61648 (2)	M6	Shihezi
		60083, 60085, 60129, 60141, 60142 (5)	M6	Ganzhou
		63431 (1)	X9	Zhanjiang
57	67023	67023, 67026 (2)	M6	Zhanjiang
58	63569	63610, 63615, 63569 (3)	M6	Weifang
		61439 (1)	X9	Shaoyang
		60145, 60151 (2)	X9	Ganzhou
		60138 (1)	M6	Ganzhou
		63351, 63600, 63609 (3)	X9	Hezhou
		63332, 63339, 63349 (3)	X9	Zhanjiang
		61866, 61867, 61869, 61875, 61877, 61878, 61889 (7)	M6	Zhanjiang
59	63560	61312 (1)	X9	Kaifeng
		61322-1, 61322-2, 61323, 61327, 61329, 61337, 61339, 61341 (8)	M6	Kaifeng

		61297, 62176, 62184, 61919, 61296, 63299, 63560, 63583, 63618 (9)	X9	Hezhou
		62163, 62172, 62188, 62191, 62202, 62209 (6)	M6	Hezhou
		61817 (1)	X9	Zhanjiang
		61867-2, 61893 (2)	M6	Zhanjiang
60	63323	61821, 63323, 63369 (3)	X9	Zhanjiang
61	63462	61990 (1)	M6	Haerbin
		63462 (1)	X9	Zhanjiang
62	67006	67006 (1)	X9	Zhanjiang
		67031 (1)	M6	Zhanjiang
63	61732	61732 (1)	X9	Kunming
64	63333	63296, 63301 (2)	X9	Zhanjiang
		62204, 62208, 63294, 63333 (4)	M6	Zhanjiang
65	63558	64558 (1)	X9	Hezhou
66	60146	60036, 60146 (2)	X9	Ganzhou
		60116 (1)	M6	Ganzhou
67	60312	60312 (1)	X9	Shaoyang
68	60341	60341 (1)	X9	Shaoyang
69	63466	63466 (1)	X9	Zhanjiang
70	60344	60344 (1)	M6	Shaoyang
71	63705	63705 (1)	X9	Fenyang
72	63709	63709 (1)	X9	Fenyang
73	63793	63793 (1)	M6	Fenyang
74	63673	63673 (1)	M6	Fenyang
75	63702	63702 (1)	M6	Fenyang
76	61243	61243 (1)	M6	Guiyang
77	61345	61345, 61346, 61349, 61351, 61352, 61353, 61354, 61355, 61356 (9)	X9	Danzhou
78	61359	61359 (1)	X9	Danzhou
79	61358	61358 (1)	X9	Danzhou
80	61380	61347, 61363 (2)	X9	Danzhou
		61380, 61381, 61383, 61394, 61396, 61397, 61403-2 (7)	X9	Danzhou
81	61627	61585, 61590, 61591, 61592, 61594, 61597, 61600, 61602, 61603, 61608, 61609, 61610, 61611, 61613, 61614, 61616, 61619, 61620, 61621, 61624, 61625, 61626, 61627 (23)	X9	Hefei
		61652, 61653, 61654, 61655, 61657, 61658, 61659, 61660, 61661, 61664,	M6	Hefei

		61665, 61666, 61668, 61669, 61670, 61673, 61674, 61675, 61678-1, 61678-2, 61679, 61682, 61683, 61684, 61687, 61690, 61691, 61692, 61693, 61694, 61695, 61696 (32)		
82	63350	63350 (1)	X9	Zhanjiang
83	61751	61751 (1)	M6	Kunming
84	61517	61517 (1)	H33	Yantai

Table S4 A detailed list of accession numbers of housekeeping genes and symbiotic gene sequences obtained in this study.

Representative strain	*recA*	*dnaK*	*glnII*	*gyrB*	*rpoB*	*nodC*	*nifH*	Nodulation
60070	PQ008258	PQ049500	PQ181574	PQ049414	PQ058151	PQ226903	PQ226822	+[a]
60073	PQ008259	PQ049501	PQ181575	PQ049415	PQ058152	PQ226904	PQ226823	+
60117	PQ008260	PQ049502	PQ181576	PQ049416	PQ058153	PQ226905	PQ226824	+
60132	PQ008261	PQ049503	PQ181577	PQ049417	PQ058154	PQ226906	PQ226825	+
60146	PQ008262	PQ049504	PQ181578	PQ049418	PQ058155	PQ226907	PQ226826	+
60192	PQ008263	PQ049505	PQ181579	PQ049419	PQ058156	PQ226908	PQ226827	+
60312	PQ008274	PQ049506	PQ181580	PQ049420	PQ058157	PQ226909	PQ226828	+
60315	PQ008264	PQ049507	PQ181581	PQ049421	PQ058158	PQ226910	PQ226829	+
60329	PQ008265	PQ049508	PQ181582	PQ049422	PQ058159	PQ226911	PQ226830	+
60333	PQ008266	PQ049509	PQ181583	PQ049423	PQ058160	PQ226912	PQ226831	+
60341	PQ008267	PQ049510	PQ181584	PQ049424	PQ058161	--[b]	--	--
60344	PQ008268	PQ049511	PQ181585	PQ049425	PQ058162	PQ226913	PQ226832	+
60346	PQ008269	PQ049512	PQ181586	PQ049426	PQ058163	PQ226914	PQ226833	+
60347	PQ008270	PQ049513	PQ181587	PQ049427	PQ058164	PQ226915	PQ226834	+
60375	PQ008271	PQ049514	PQ181588	PQ049428	PQ058165	PQ226916	PQ226835	+
60458	PQ008273	PQ049516	PQ181590	PQ049430	PQ058167	PQ226917	PQ226836	+
60461	PQ014276	PQ049517	PQ181591	PQ049431	PQ058168	PQ226918	PQ226837	+
60507	PQ014277	PQ049518	PQ181592	PQ049432	PQ058169	PQ226919	PQ226838	+
60519	PQ014278	PQ049519	PQ181593	PQ049433	PQ058170	PQ226920	PQ226839	+
60522	PQ014279	PQ049520	PQ181594	PQ049434	PQ058171	PQ226921	PQ226840	+
60524	PQ014280	PQ049521	PQ181595	PQ049435	PQ058172	PQ226922	PQ226841	+
60531	PQ014281	PQ049522	PQ181596	PQ049436	PQ058173	PQ226923	PQ226842	+
60533	PQ014282	PQ049523	PQ181597	PQ049437	PQ058174	PQ226924	PQ226843	+

60535	PQ014283	PQ049524	PQ181598	PQ049438	PQ058175	PQ226925	PQ226844	+
61243	PQ014285	PQ049526	PQ181600	PQ049440	PQ058177	--	--	--
61250	PQ014286	PQ049527	PQ181601	PQ049441	PQ058178	--	--	--
61345	PQ014287	PQ049528	PQ181602	PQ049442	PQ058179	PQ226926	PQ226845	+
61358	PQ014288	PQ049529	PQ181603	PQ049443	PQ058180	PQ226927	PQ226846	+
61359	PQ014289	PQ049530	PQ181604	PQ049444	PQ058181	PQ226928	PQ226847	+
61380	PQ014290	PQ049531	PQ181605	PQ049445	PQ058182	PQ226929	PQ226848	+
61517	PQ014291	PQ049532	PQ211146	PQ049446	PQ058183	PQ226930	PQ226849	+
61544	PQ014292	PQ049533	PQ181606	PQ049447	PQ058184	PQ226931	PQ226850	+
61627	PQ014293	PQ049534	PQ181607	PQ049448	PQ058185	PQ226932	PQ226851	+
61638	PQ014294	PQ049535	PQ181608	PQ049449	PQ058186	PQ226933	PQ226852	+
61707	PQ014295	PQ049536	PQ181609	PQ049450	PQ058187	PQ226934	PQ226853	+
61720	PQ014296	PQ049537	PQ181610	PQ049451	PQ058188	PQ226935	PQ226854	+
61723	PQ014344	PQ049585	PQ181657	PQ049499	PQ058236	PQ226936	PQ226855	+
61727	PQ014297	PQ049538	PQ181611	PQ049452	PQ058189	PQ226937	PQ226856	+
61732	PQ014298	PQ049539	PQ181612	PQ049453	PQ058190	PQ226938	PQ226857	+
61733	PQ014299	PQ049540	PQ181613	PQ049454	PQ058191	PQ226939	PQ226858	+
61734	PQ014300	PQ049541	PQ181614	PQ049455	PQ058192	PQ226940	PQ226859	+
61737	PQ014301	PQ049542	PQ181615	PQ049456	PQ058193	PQ226941	PQ226860	+
61743	PQ014302	PQ049543	PQ181616	PQ049457	PQ058194	PQ226942	PQ226861	+
61751	PQ014303	PQ049544	PQ181617	PQ049458	PQ058195	PQ226943	PQ226862	+
61756	PQ014304	PQ049545	PQ181618	PQ049459	PQ058196	PQ226944	PQ226863	+
61862	PQ014305	PQ049546	PQ181619	PQ049460	PQ058197	PQ226945	PQ226864	+
61887	PQ014306	PQ049547	PQ181620	PQ049461	PQ058198	PQ226946	PQ226865	+
61890	PQ014307	PQ049548	PQ181621	PQ049462	PQ058199	PQ226947	PQ226866	+

61978	PQ014308	PQ049549	PQ181622	PQ049463	PQ058200	PQ226948	PQ226867	+
62056	PQ014309	PQ049550	PQ181623	PQ049464	PQ058201	PQ226949	PQ226868	+
62104	PQ014310	PQ049551	PQ181624	PQ049465	PQ058202	PQ226950	PQ226869	+
62186	PQ014311	PQ049552	PQ211147	PQ049466	PQ058203	PQ226951	PQ226870	+
62198	PQ014312	PQ049553	PQ181625	PQ049467	PQ058204	PQ226952	PQ226871	+
62286	PQ014313	PQ049554	PQ181626	PQ049468	PQ058205	PQ226953	PQ226872	+
63323	PQ014314	PQ049555	PQ181627	PQ049469	PQ058206	PQ226954	PQ226873	+
63333	PQ014315	PQ049556	PQ181628	PQ049470	PQ058207	PQ226955	PQ226874	+
63350	PQ014316	PQ049557	PQ181629	PQ049471	PQ058208	PQ226956	PQ226875	+
63431	PQ014317	PQ049558	PQ181630	PQ049472	PQ058209	PQ226957	PQ226876	+
63462	PQ014318	PQ049559	PQ181631	PQ049473	PQ058210	PQ226958	PQ226877	+
63466	PQ014319	PQ049560	PQ181632	PQ049474	PQ058211	PQ226959	PQ226878	+
63479	PQ014320	PQ049561	PQ181633	PQ049475	PQ058212	PQ226960	PQ226879	+
63514	PQ014321	PQ049562	PQ181634	PQ049476	PQ058213	PQ226961	PQ226880	+
63531	PQ014322	PQ049563	PQ181635	PQ049477	PQ058214	PQ226962	PQ226881	+
63542	PQ014323	PQ049564	PQ181636	PQ049478	PQ058215	PQ226963	PQ226882	+
63558	PQ014324	PQ049565	PQ181637	PQ049479	PQ058216	PQ226964	PQ226883	+
63559	PQ014325	PQ049566	PQ181638	PQ049480	PQ058217	PQ226965	PQ226884	+
63560	PQ014326	PQ049567	PQ181639	PQ049481	PQ058218	PQ226966	PQ226885	+
63569	PQ014327	PQ049568	PQ181640	PQ049482	PQ058219	PQ226967	PQ226886	+
63607	PQ014328	PQ049569	PQ181641	PQ049483	PQ058220	PQ226968	PQ226887	+
63614	PQ014329	PQ049570	PQ181642	PQ049484	PQ058221	--	PQ226888	--
63664	PQ014330	PQ049571	PQ181643	PQ049485	PQ058222	--	PQ226889	--
63673	PQ014331	PQ049572	PQ181644	PQ049486	PQ058223	PQ226969	PQ226890	+
63702	PQ014332	PQ049573	PQ181645	PQ049487	PQ058224	PQ226970	PQ226891	+

63703	PQ014333	PQ049574	PQ181646	PQ049488	PQ058225	PQ226971	PQ226892	+
63705	PQ014334	PQ049575	PQ181647	PQ049489	PQ058226	PQ226972	PQ226893	+
63709	PQ014335	PQ049576	PQ181648	PQ049490	PQ058227	PQ226973	PQ226894	+
64002	PQ014336	PQ049577	PQ181649	PQ049491	PQ058228	PQ226974	PQ226895	+
64004	PQ014337	PQ049578	PQ181650	PQ049492	PQ058229	PQ226975	PQ226896	+
64010	PQ014338	PQ049579	PQ181651	PQ049493	PQ058230	PQ226976	PQ226897	+
64011	PQ014339	PQ049580	PQ181652	PQ049494	PQ058231	PQ226977	PQ226898	+
64103	PQ014340	PQ049581	PQ181653	PQ049495	PQ058232	PQ226978	PQ226899	+
66012	PQ014341	PQ049582	PQ181654	PQ049496	PQ058233	PQ226979	PQ226900	+
67006	PQ014342	PQ049583	PQ181655	PQ049497	PQ058234	PQ226980	PQ226901	+
67023	PQ014343	PQ049584	PQ181656	PQ049498	PQ058235	PQ226981	PQ226902	+

[a]+ means formed nodules on peanut root; [b]-- means failed in amplification of the genes or without nodulation abilities on peanut.

Table S5 A detailed list of accession numbers of housekeeping genes of reference type strains downloaded from GenBank.

Representative strain	*recA*	*dnaK*	*glnII*	*gyrB*	*rpoB*
B. guangxiense CCBAU53363^{T}	KC509279	KC508974	KC509033	KC509082	KC509328
B. daqingense CCBAU 15774^{T}	HQ231270	KF962684	HQ231301	JX437669	JX437676
B. stylosanthis BR446^{T}	KU724163	KU724145	KU724148	KU724151	KU724166
B. huanghuaihaiense CCBAU23303^{T}	HM107234	KF962686	HQ231639	JX437672	JX437679
B. canariense LMG22265^{T}	AY591553	AY923047	AY386765	FM253220	FM253263
B. betae PL7HG1^{T}	AB353734	AY923046	AB353733	AB353735	GU562860
B. cytisi LMG25866^{T}	JN186293	JQ945184	GU001594	JN186292	JN186288
B. rifense CTAW71^{T}	GU001585	JQ945187	GU001604	KC569466	KC569468
B. kavangense 14-3^{T}	KM378399	KR259949	KM378446	KX661397	KM378311
B. subterraneum 582-1^{T}	KM378397	KP308157	KM378484	KX661396	KM378349
B. vignae 7-2^{T}	KM378374	KR259951	KM378443	GCA 004114425	KM378308
B. centrolobii BR10245^{T}	KX527954	KX527928	KX527991	LUUB01000079	KF983827
B. neotropicale BR10247^{T}	KJ661714	KJ661693	KJ661700	KJ661707	KF983829
B. ingae BR 10250^{T}	KF927061	KF927055	KF927067	KF927079	KF927073
B. iriomotense LMG24129^{T}	AB300996	JF308944	AB300995	HQ873308	HQ587646
B. manausense BR3351^{T}	KF785992	KF786001	KF785986	KF786000	KF785998
B. denitrificans LMG8443^{T}	FM253196	FM253325	HM047121	FM253239	FM253282
B. oligotrophicum LMG10732^{T}	JQ619231	AP012603	JQ619233	KC569467	KF962713
B. lablabi CCBAU 23086^{T}	GU433522	KF896185	GU43349	JX437670	JX437677
B. jicamae LMG24556^{T}	HM047133	JN207408	FJ428204	HQ873309	HQ587647
B. paxllaeri LMTR21^{T}	JX943617	AY923038	KF896169	KF896195	KP308154
B. icense LMTR13^{T}	JX943615	KF896182	KF896175	KF896201	GENOME
B. retamae Ro19^{T}	KC247094	KF896184	KC247108	KF962698	KF962714
B. valentinum LmjM3^{T}	JX518589	GCA001440405	JX518575	GCA001440405	GCA001440405
B. namibiense 5-10^{T}	KM378377	KP402058	KM378440	KX661393	KM378306
B. macuxiense BR10303^{T}	KX527958	KX527932	KX527995	KX528008	KX527969
B. embrapense SEMIA6208^{T}	HQ634899	KP234519	GQ160500	HQ63489	GENOME
B. mercantei SEMIA 6399^{T}	KX690615	KX690617	KX690621	KX690623	GCA 01982635
B. tropiciagri SEMIA6148^{T}	FJ391168	FJ391008	FJ391048	HQ634890	GENOME
B. viridifuturi SEMIA690^{T}	KR149140	KR149128	KR149131	KR149134	GENOME
B. elkanii USDA 76^{T}	AY591568	AM168363	AY599117	AB07058	EF190188
B. pachyrhizi LMG24246^{T}	HM047130	JF308946	FJ428201	HQ873310	HQ587648
B. ferriligni CCBAU51502^{T}	KJ818112	MG811657	KJ818099	KJ818102	MG811655
B. ottawaense OO85^{T}	HQ587287	JF308816	HQ587750	HQ873165	HQ587518
B. nanningense CCBAU 53390^{T}	KC509274	KC508969	KC509028	KC509085	KC509323
B.liaoningense USDA3622^{T}	AY494833	AY923041	AY386775	FM253223	FM253266
B. arachidis CCBAU 051107^{T}	KF962707	KJ560556	HM107251	JX437675	JX437682
B. yuanmingense CCBAU 10071^{T}	AY591566	AY923039	AY386780	FM253226	FM253269

B. diazoefficiens USDA 110^T	NC004463	BA000040	CP011360	BA000040	BA000040
B.japonicum USDA6^T	AM168341	AM168362	HQ587875	AB070586	AM295349
B. guangdongense CCBAU 51649^T	KC509269	KC508964	KC509023	KC509072	KC509318
B. guangzhouense CCBAU 53424^T	KC509282	KC508977	KC509036	KC509085	KC509331

Table S6 MLSA similarities of representative strains with each genospecies.

Species	Representative strains	MLSA similarities with most related type strain	MLSA similarities among the representative strains	Number of isolates	Number of distribution sites	Haplotype of *recA*
B. arachidis	61734, 62286	*B. arachidis* CCBAU 051107^T, 97.3-99.5%	97.3%	5	2	29, 30
B. diazoefficiens	60346, 60347	*B. diazoefficiens* USDA 110^T, 97.3%	97.5%	2	2	45, 46
B. ferriligni	61517	*B. ferriligni* CCBAU51502^T, 97.8%	--	1	1	84
B. guangdongense	62198, 63431, 67023, 63569	*B. guangdongense* CCBAU 51649^T, 98.8-99.4%	98.2-99.5%	60	8	55 - 58
B. guangzhouense	60344, 63466	*B. guangzhouense* CCBAU 53424^T, 99.5-99.7%	99.5%	2	2	69, 70
B. japonicum	60315, 61707, 61723	*B. japonicum* USDA6^T, 97.2-98.8%	97.6-98.0%	11	3	47, 48, 49
B. liaoningense	62056, 62104, 60117, 61978, 60192, 60519, 60507, 60533	*B. liaoningense* USDA 3622^T, 99.2-98.1%	97.4-99.9%	220	12	15 - 22
B. manausense	61732	*B. manausense* BR3351^T, 97.5%	--	1	1	63
B. nanningense	60458, 61890	*B. nanningense* CCBAU 53390^T, 98.8-99.6%	99.1%	59	5	9, 10
B. ottawaense	64011, 64002, 61743, 64010	*B. ottawaense* OO99^T, 99.0-99.6%	98.1-99.5%	220	7	1 - 4
B. stylosanthis	60461	*B. stylosanthis* BR 446^T, 98.3%	--	2	1	42
B. yuanmingense	60333, 63531, 61733, 61638, 63479, 63514	*B. yuanmingense* CCBAU 10071^T, 97.1-99.9%	97.5-99.7%	177	8	36 - 41
Bradyrhizobium sp. I	61544	*B. ottawaense* OO99^T, 95.6%	--	1	1	5
Bradyrhizobium sp. II	64004	*B. ottawaense* OO99T, 95.8%	--	5	3	6
Bradyrhizobium sp. III	60329	*B. liaoningense* USDA 3622^T, 95.6%	--	2	2	7
Bradyrhizobium sp. IV	60375	*B. nanningense* CCBAU 53390^T, 96.4%	--	1	1	8
Bradyrhizobium sp. V	60070	*B. liaoningense* USDA 3622^T, 95.0%	--	1	1	11
Bradyrhizobium sp. VI	60132, 61887, 61862	*B. yuanmingense* CCBAU 10071^T, 96.4%	98.4-98.8%	25	2	12, 13, 14
Bradyrhizobium sp. VII	60522, 60524, 60531	*B. daqingense* CCBAU 15774^T, 95.8-95.9%	99.2-99.5%	34	2	23, 24, 25

Bradyrhizobium sp.VIII	60535	*B. daqingense* CCBAU 15774^T, 96.5-96.8%	--	2	1	26
Bradyrhizobium sp. IX	63607	*B. arachidis* CCBAU 051107^T, 95.8%	--	2	2	27
Bradyrhizobium sp. X	61756	*B. arachidis* CCBAU 051107^T, 96.0%	--	1	1	28
Bradyrhizobium sp. XI	61720	*B. huanghuaihaiense* CCBAU 23303^T, 95.8%	--	3	2	31
Bradyrhizobium sp. XII	63542, 63559, 64103, 66012	*B. yuanmingense* CCBAU 10071^T, 96.5-97.0%	97.6-99.7%	14	6	32 - 35
Bradyrhizobium sp. XIII	61250	*B. stylosanthis* BR 446^T, 96.7%	--	1	1	43
Bradyrhizobium sp. XIV	63614	*B. huanghuaihaiense* CCBAU 23303^T, 96.6%	--	1	1	44
Bradyrhizobium sp. XV	60073	*B. diazoefficiens* USDA 110^T, 95.1%	--	1	1	50
Bradyrhizobium sp. XVI	61727, 61737	*B. rifense* CTAW71^T, 96.2%	98.4%	7	1	51, 52
Bradyrhizobium sp. XVII	62186	*B. vignae* 7-2^T, 95.2%	--	10	2	53
Bradyrhizobium sp. XVIII	63664	*B. liaoningense* USDA 3622^T, 96.0%	--	1	1	54
Bradyrhizobium sp. XIX	63560	*B. guangdongense* CCBAU 51649^T, 96.7%	--	27	3	59
Bradyrhizobium sp. XX	63323	*B. guangdongense* CCBAU 51649^T, 96.4%	--	3	1	60
Bradyrhizobium sp. XXI	63462, 67006	*B. stylosanthis* BR 446^T, 94.6-95.0%	98.2%	4	2	61, 62
Bradyrhizobium sp. XXII	63333	*B. guangdongense* CCBAU 51649^T, 94.5%	--	6	1	64
Bradyrhizobium sp. XXIII	63558	*B. guangdongense* CCBAU 51649^T, 94.3%	--	1	1	65
Bradyrhizobium sp. XXIV	60146	*B. guangdongense* CCBAU 51649^T, 94.9%	--	3	1	66
Bradyrhizobium sp. XXV	60312	*B. guangdongense* CCBAU 51649^T, 93.7%	--	1	1	67
Bradyrhizobium sp. XXVI	60341	*B. manausense* BR3351^T, 95.6%	--	1	1	68
Bradyrhizobium sp. XXVII	63705, 63709, 63793, 63673, 63702	*B. lablabi* CCBAU 23086^T, 95.7-96.4%	98.5-99.4	5	1	71 - 75
Bradyrhizobium sp. XXVIII	61243	*Bradyrhizobium elkanii* USDA 76^T, 94.0%	--	1	1	76
Bradyrhizobium sp. XXIX	61345, 61358, 61359, 61380, 61627, 63350	*B. elkanii* USDA 76^T, 94.8-95.3%	97.8-99.9%	76	3	77 - 82
Bradyrhizobium sp. XXX	61751	*B. tropiciagri* SEMIA 6148^T, 95.2%	--	1	1	83

Table S7 The geographic distribution of peanut rhizobium genospecies.

Genospecies	1[a]	2	3	4	5	6	7	8	9	10	11	12	13	14	15	16	17	18	19	20
B. ottawaense	0[b]	72	103	0	0	0	0	0	23	6	6	8	0	0	0	0	2	0	0	0
Bradyrhizobium sp. I	0	0	0	0	0	0	0	0	0	1	0	0	0	0	0	0	0	0	0	0
Bradyrhizobium sp. II	0	0	1	0	0	0	0	0	0	3	0	0	1	0	0	0	0	0	0	0
Bradyrhizobium sp. III	0	0	0	0	0	0	0	1	0	0	0	1	0	0	0	0	0	0	0	0
Bradyrhizobium sp. IV	0	0	0	0	0	0	0	0	0	0	0	1	0	0	0	0	0	0	0	0
B. nanningense	0	0	0	0	0	0	0	9	0	27	0	18	4	0	0	0	0	0	1	0
Bradyrhizobium sp. V	0	0	0	0	0	0	0	0	0	0	0	0	1	0	0	0	0	0	0	0
Bradyrhizobium sp. VI	0	0	0	0	0	0	0	0	0	0	0	0	15	0	0	0	0	0	10	0
B. liaoningense	24	1	2	0	0	0	20	0	34	29	13	13	22	1	19	0	0	42	0	0
Bradyrhizobium sp. VII	0	0	0	0	0	0	0	0	0	0	7	0	0	0	27	0	0	0	0	0
Bradyrhizobium sp. VIII	0	0	0	0	0	0	0	0	0	0	0	0	0	0	2	0	0	0	0	0
Bradyrhizobium sp. IX	0	0	0	0	0	0	0	0	0	0	0	0	0	1	0	0	0	1	0	0
Bradyrhizobium sp. X	0	0	0	0	0	0	0	0	0	0	0	0	0	0	0	0	1	0	0	0
B. arachidis	0	0	0	0	0	0	0	0	4	0	0	0	0	0	0	0	1	0	0	0
Bradyrhizobium sp. XI	0	2	0	0	0	0	0	0	0	0	0	0	0	0	0	0	1	0	0	0
Bradyrhizobium sp. XII	0	5	1	0	1	0	0	0	2	0	0	2	0	0	0	0	0	3	0	0
B. yuanmingense	0	0	0	10	66	0	0	46	0	2	8	18	4	0	0	0	23	0	0	0
B. stylosanthis	0	0	0	0	0	0	0	0	0	0	0	2	0	0	0	0	0	0	0	0
Bradyrhizobium sp. XIII	0	0	0	0	0	0	0	0	0	0	0	0	0	0	0	1	0	0	0	0
Bradyrhizobium sp. XIV	0	0	0	0	0	0	0	1	0	0	0	0	0	0	0	0	0	0	0	0
B. diazoefficiens	0	1	0	0	0	0	0	0	0	0	0	0	0	1	0	0	0	0	0	0
B. japonicum	0	8	0	0	0	0	0	0	0	0	0	0	0	1	0	0	2	0	0	0
Bradyrhizobium sp. XV	0	1	0	0	0	0	0	0	0	0	0	0	0	0	0	0	0	0	0	0

Bradyrhizobium sp. XVI	0	0	0	0	0	0	0	0	0	0	0	0	0	0	0	0	7	0	0	0
Bradyrhizobium sp. XVII	0	0	0	0	0	0	0	0	0	0	0	0	0	0	0	0	0	7	0	3
Bradyrhizobium sp. XVIII	0	0	0	0	1	0	0	0	0	0	0	0	0	0	0	0	0	0	0	0
B. guangdongense	0	0	0	2	0	0	0	3	0	0	0	0	18	1	0	12	0	10	13	1
Bradyrhizobium sp. XIX	0	0	0	0	0	0	0	0	0	0	9	0	0	0	0	0	0	15	3	0
Bradyrhizobium sp. XX	0	0	0	0	0	0	0	0	0	0	0	0	0	0	0	0	0	0	3	0
Bradyrhizobium sp. XXI	1	0	0	0	0	0	0	0	0	0	0	0	0	0	0	0	0	0	3	0
B. manausens	0	0	0	0	0	0	0	0	0	0	0	0	0	0	0	0	1	0	0	0
Bradyrhizobium sp. XXII	0	0	0	0	0	0	0	0	0	0	0	0	0	0	0	0	0	0	6	0
Bradyrhizobium sp. XXIII	0	0	0	0	0	0	0	0	0	0	0	0	0	0	0	0	0	1	0	0
Bradyrhizobium sp. XXIV	0	0	0	0	0	0	0	0	0	0	0	0	3	0	0	0	0	0	0	0
Bradyrhizobium sp. XXV	0	0	0	0	0	0	0	0	0	0	0	0	0	1	0	0	0	0	0	0
Bradyrhizobium sp. XXVI	0	0	0	0	0	0	0	0	0	0	0	0	0	1	0	0	0	0	0	0
B. guangzhouense	0	0	0	0	0	0	0	0	0	0	0	0	0	1	0	0	0	0	1	0
Bradyrhizobium sp. XXVII	0	0	0	0	0	5	0	0	0	0	0	0	0	0	0	0	0	0	0	0
Bradyrhizobium sp. XXVIII	0	0	0	0	0	0	0	0	0	0	0	0	0	0	0	1	0	0	0	0
Bradyrhizobium sp. XXIX	0	0	0	0	0	0	0	0	0	0	0	55	0	0	0	0	0	0	1	20
Bradyrhizobium sp. XXX	0	0	0	0	0	0	0	0	0	0	0	0	0	0	0	0	1	0	0	0
B. ferriligni	0	0	0	0	0	0	0	0	0	1	0	0	0	0	0	0	0	0	0	0

[a]1, Haerbin; 2, Siping; 3, Fuxin; 4, Shihezi; 5, Baoding; 6, Fenyang; 7, Lanzhou; 8, Weifang; 9, Laixi; 10, Yantai; 11, Kaifeng; 12, Hefei; 13, Ganzhou; 14, Shaoyang; 15, Nanchong; 16, Guiyang; 17, Kunming; 18, Hezhou; 19, Zhanjiang; 20, Danzhou; [b], the isolates' number of the corresponding genospecies.

Table S8 The detailed Canoco results of soil characteristics and genospecies.

Name	Explains %	Contribution %	pseudo-F	*P*
AP	12.3	22.5	2.4	0.026
pH	10.5	19.2	2.2	0.018
AN	6.8	12.5	1.5	0.158
OC	4.5	8.2	1.0	0.490
TN	9.2	16.7	2.1	0.058
AK	6.3	11.6	1.5	0.162
EC	5.2	9.5	1.3	0.244

Table S9 The detailed Canoco results of climate variables and peanut rhizobium genospecies.

Name	Explains %	Contribution %	pseudo-F	*P*
MAT	13.5	41.8	2.7	0.002
MAP	9.4	29.1	2.0	0.04
TS	6.0	18.6	1.3	0.276
PS	3.4	10.5	0.7	0.672

Table S10 The tolerance range of soil environmental factors for each peanut rhizobium genospecies.

Genospecies	AK (mg/kg)	AP (mg/kg)	AN (mg/kg)	TN%	OC%	EC (μs/cm)	pH	MAP （mL）	MAT (℃)	PS	TS
B. ottawaense	199.2±5.1-363.8±5.3	13.3±2.3-82.8±1.3	38.3±2.8-141.2±2.5	0.079±0.021-0.139±0.043	0.753±0.116-1.178±0.362	52.8±4.5-169.5±8.9	5.85±0.21-8.31±0.13	503-1146	7.8-14.9	0.52-1.1	0.29-1.57
Bradyrhizobium sp. I	237.2±2.8	20.6±3.2	38.5±4.3	0.079±0.021	0.753±0.116	104.0±6.5	7.66±0.29	698	12	0.93	0.78
Bradyrhizobium sp. II	199.4±2.7-239.5±3.2	20.6±3.2-82.8±1.3	38.5±4.3-119.0±5.6	0.079±0.021-0.134±0.012	0.753±0.116-1.145±0.008	69.4±3.6-104.0±6.5	5.81±0.32-7.66±0.29	503-1492	7.8-18.9	0.5-1.1	0.38-1.57
Bradyrhizobium sp. III	315.1±6.6-395.6±4.3	27.2±3.4-32.0±2.3	38.3±2.8-73.1±1.8	0.104±0.019-0.139±0.043	1.041±0.108-1.089±0.108	92.1±4.8-172.9±2.8	5.85±0.21-7.99±0.17	690-1146	13-16	0.52-1.06	0.55-0.77
Bradyrhizobium sp. IV	315.1±6.6	27.2±3.4	38.3±2.8	0.139±0.043	1.041±0.108	92.1±4.8	5.85±0.21	1146	16	0.52	0.55
B. nanningense	199.4±2.7-395.6±4.3	14.0±1.8-41.1±1.5	38.3±2.8-119.0±5.6	0.079±0.021-0.166±0.053	0.753±0.116-1.823±0.026	70.6±7.7-172.9±2.8	5.85±0.21-7.99±0.17	690-1763	12-23.3	0.5-1.06	0.21-0.78
Bradyrhizobium sp. V	199.4±2.7	41.1±1.5	119.0±5.6	0.134±0.012	1.043±0.072	70.6±7.7	5.81±0.32	1492	18.9	0.5	0.38
Bradyrhizobium sp. VI	199.4±2.7	41.1±1.5	119.0±5.6	0.134±0.012	1.043±0.072	70.6±7.7	5.81±0.32	1492	18.9	0.5	0.38
B. liaoningense	199.4±2.7-437.0±2.9	11.0±0.8-82.8±1.3	38.3±2.8-122.2±6.8	0.079±0.021-407±0.048	0.753±0.116-4.561±0.630	52.8±4.5-288.0±4.3	4.92±0.23-8.31±0.13	345-1567	2.2-20.1	0.47-1.1	0.33-0.83
Bradyrhizobium sp. VII	228.6±4.3-314.0±3.2	29.7±0.8-42.5±2.8	61.8±1.8-98.7±3.6	0.104±0.028-0.153±0.008	0.934±0.237-1.101±0.211	106.2±5.5-160.7±3.3	7.96±0.09-8.31±0.13	628-1090	14.6-17.5	0.78-0.87	0.41-0.65
Bradyrhizobium sp. VIII	228.6±4.3	29.7±0.8	98.7±3.6	0.153±0.008	1.101±0.211	106.2±5.5	7.96±0.09	1090	17.5	0.78	0.41
Bradyrhizobium sp. IX	294.4±3.6-412.2±6.8	34.2±2.1-46.3±2.5	71.8±6.4-122.2±6.8	0.180±0.046-0.407±0.048	1.002±0.009-4.561±0.630	70.2±1.2-134.1±2.7	4.92±0.23-5.68±0.17	1353-1567	17.3-20.1	0.47-0.59	0.33-0.46
Bradyrhizobium sp. X	199.2±5.1	13.3±2.3	141.2±2.5	0.117±0.036	0.844±0.097	169.5±8.9	6.10±0.35	928	14.9	0.81	0.29
B. arachidis	199.2±5.1-291.8±1.9	13.3±2.3-32.9±0.7	58.3±3.5-141.2±2.5	0.080±0.010-0.117±0.036	0.754±0.093-0.844±0.097	139.4±7.4-169.5±8.9	6.10±0.35-6.95±0.04	698-928	12.8-14.9	0.81-0.92	0.29-0.73
Bradyrhizobium sp. XI	199.2±5.1-363.8±5.3	13.3±2.3-37.5±1.8	102.9±2.3-141.2±2.5	0.117±0.036-0.122±0.007	0.844±0.097-1.178±0.362	52.8±4.5-169.5±8.9	6.10±0.35-8.22±0.37	618-928	8.1-14.9	0.81-1.04	0.29-14.8
Bradyrhizobium sp. XII	239.5±3.2-412.2±6.8	22.4±2.3-82.8±1.3	37.1±1.4-122.2±6.8	0.080±0.010-0.407±0.048	0.754±0.093-4.561±0.630	52.8±4.5-139.4±7.4	5.68±0.17-8.22±0.37	503-1567	7.8-20.1	0.52-1.29	0.33-1.57
B. yuanmingense	199.2±5.1-395.6±4.3	13.3±2.3-42.5±2.8	26.8±3.6-141.2±2.5	0.071±0.017-0.139±0.043	0.844±0.097-2.910±0.006	70.6±7.7-172.9±2.8	5.81±0.32-8.31±0.13	153-1492	8.3-18.9	0.5-1.29	0.29-1.7
B. stylosanthis	315.1±6.6	27.2±3.4	38.3±2.8	0.139±0.043	1.041±0.108	92.1±4.8	5.85±0.21	1146	16	0.52	0.55
Bradyrhizobium sp. XIII	264.4±5.9	10.1±1.6	94.9±1.7	0.252±0.025	2.603±0.139	198.0±8.6	7.12±0.17	1113	15.2	0.68	0.43
Bradyrhizobium sp. XIV	395.6±4.3	32.0±2.3	73.1±1.8	0.104±0.019	1.089±0.108	172.9±2.8	7.99±0.17	690	13	1.06	0.77
B. diazoefficiens	294.4±3.6-363.8±5.3	37.5±1.8-46.3±2.	71.8±6.4-102.9±2.3	0.122±0.007-0.180±0.046	1.002±0.009-1.178±0.362	52.8±4.5-134.1±2.7	4.92±0.23-8.22±0.37	618-1353	8.1-17.3	0.47-1.04	0.46-1.48
B. japonicum	199.2±5.1-363.8±5.3	13.3±2.3-46.3±2.	71.8±6.4-141.2±2.5	0.117±0.036-0.180±0.046	0.844±0.097-1.178±0.362	52.8±4.5-169.5±8.9	4.92±0.23-8.22±0.37	618-1353	8.1-17.3	0.47-1.04	0.29-1.48
Bradyrhizobium sp. XV	363.8±5.3	37.5±1.8	102.9±2.3	0.122±0.007	1.178±0.362	52.8±4.5	8.22±0.37	618	8.1	1.04	1.48
Bradyrhizobium sp. XVI	199.2±5.1	13.3±2.3	141.2±2.5	0.117±0.036	0.844±0.097	169.5±8.9	6.10±0.35	928	14.9	0.81	0.29
Bradyrhizobium sp. XVII	259.1±2.7-	12.9±2.6-	73.2±1.9-	0.183±0.009-	1.671±0.139-	70.2±1.2-72.1±4.3	4.92±0.18-	1567-	20.1-	0.59-	0.15-

	412.2±6.8	34.2±2.1	122.2±6.8	0.407±0.048	4.561±0.630		5.68±0.17	1708	24.1	0.74	0.33
Bradyrhizobium sp. XVIII	251.1±3.2	22.4±2.3	37.1±1.4	0.123±0.076	2.910±0.006	131.3±6.1	8.11±0.08	507	13.2	1.29	0.8
B. guangdongense	199.4±2.7-412.2±6.8	10.1±1.6-46.3±2.5	26.8±3.6-122.2±6.8	0.071±0.017-0.407±0.048	1.002±0.009-4.561±0.630	70.2±1.2-198.0±8.6	4.92±0.23-8.13±0.26	153-1763	8.3-24.1	0.47-1.06	0.15-1.7
Bradyrhizobium sp. XIX	239.8±3.8-412.2±6.8	14.0±1.8-42.5±2.8	61.8±1.8-122.2±6.8	0.104±0.028-0.407±0.048	0.934±0.237-4.561±0.630	70.2±1.2-160.7±3.3	5.68±0.17-8.31±0.13	628-1763	14.6-23.3	0.59-0.87	0.21-0.65
Bradyrhizobium sp. XX	239.8±3.8	14.0±1.8	63.0±7.3	0.166±0.053	1.823±0.026	102.1±1.5	5.89±0.23	1763	23.3	0.69	0.21
Bradyrhizobium sp. XXI	239.8±3.8-437.0±2.9	14.0±1.8-41.1±3.2	63.0±7.3-94.5±1.8	0.166±0.053-0.214±0.012	1.462±0.521-1.823±0.026	102.1±1.5-170.0±6.8	5.89±0.23-6.97±0.16	530-1763	2.2-23.3	0.69-1.1	0.21-6.83
B. manausens	199.2±5.1	13.3±2.3	141.2±2.5	0.117±0.036	0.844±0.097	169.5±8.9	6.10±0.35	928	14.9	0.81	0.29
Bradyrhizobium sp. XXII	239.8±3.8	14.0±1.8	63.0±7.3	0.166±0.053	1.823±0.026	102.1±1.5	5.89±0.23	1763	23.3	0.69	0.21
Bradyrhizobium sp. XXIII	412.2±6.8	34.2±2.1	122.2±6.8	0.407±0.048	4.561±0.630	70.2±1.2	5.68±0.17	1567	20.1	0.59	0.33
Bradyrhizobium sp. XXIV	199.4±2.7	41.1±1.5	119.0±5.6	0.134±0.012	1.043±0.072	70.6±7.7	5.81±0.32	1492	18.9	0.5	0.38
Bradyrhizobium sp. XXV	294.4±3.6	46.3±2.5	71.8±6.4	0.180±0.046	1.002±0.009	134.1±2.7	4.92±0.23	1353	17.3	0.47	0.46
Bradyrhizobium sp. XXVI	294.4±3.6	46.3±2.5	71.8±6.4	0.180±0.046	1.002±0.009	134.1±2.7	4.92±0.23	1353	17.3	0.47	0.46
B. guangzhouense	239.8±3.8-294.4±3.6	14.0±1.8-46.3±2.5	63.0±7.3-71.8±6.4	0.166±0.053-0.180±0.046	1.002±0.009-1.823±0.026	102.1±1.5-134.1±2.7	4.92±0.23-5.89±0.23	1353-1763	17.3-23.3	0.47-0.69	0.21-0.46
Bradyrhizobium sp. XXVII	326.2±7.6	11.3±1.4	80.5±2.1	0.082±0.023	2.290±0.651	208.0±9.6	8.64±0.20	462	10.4	1.01	0.96
Bradyrhizobium sp. XXVIII	264.4±5.9	10.1±1.6	94.9±1.7	0.252±0.025	2.603±0.139	198.0±8.6	7.12±0.17	1113	15.2	0.68	0.43
Bradyrhizobium sp. XXIX	239.8±3.8-315.1±6.6	12.9±2.6-27.2±3.4	38.3±2.8-73.2±1.9	0.139±0.043-0.183±0.009	1.041±0.108-1.823±0.026	72.1±4.3-102.1±1.5	4.92±0.18-5.89±0.23	1146-1763	16-24.1	0.52-0.74	0.15-0.55
Bradyrhizobium sp. XXX	199.2±5.1	13.3±2.3	141.2±2.5	0.117±0.036	0.844±0.097	169.5±8.9	6.10±0.35	928	14.9	0.81	0.29
B. ferriligni	237.2±2.8	20.6±3.2	38.5±4.3	0.079±0.021	0.753±0.116	104.0±6.5	7.66±0.29	698	12	0.93	0.78

Table S11 The statistics of the isolates distributed in different sites.

Species	All[a]	NC[b]	SC[c]	X9-all	M6-all	X9-NC	M6-NC	X9-SC	M6-SC	H19	H30	H33	H29
B. arachidis	5	4	1	0	1	0	0	0	1	0	0	0	4
B. diazoefficiens	2	1	1	0	1	0	0	0	1	0	0	1	0
B. ferriligni	1	1	0	0	0	0	0	0	0	0	0	1	0
B. guangdongense	60	5	55	23	37	0	5	23	32	0	0	0	0
B. guangzhouense	2	0	2	1	1	0	0	1	1	0	0	0	0
B. japonicum	11	8	3	1	2	0	0	1	2	0	8	0	0
B. liaoningense	220	123	97	103	51	37	22	66	29	2	14	16	34
B. manausens	1	0	1	1	0	0	0	1	0	0	0	0	0
B. nanningense	59	36	23	5	9	3	6	2	3	37	2	6	0
B. ottawaense	220	210	10	57	62	49	60	8	2	32	17	29	23
B. stylosanthis	2	0	2	0	0	0	0	0	0	0	0	2	0
B. yuanmingense	177	132	45	89	69	85	55	4	14	8	9	2	0
Bradyrhizobium sp. I	1	1	0	0	0	0	0	0	0	0	1	0	0
Bradyrhizobium sp. II	5	4	1	1	1	0	1	1	0	0	3	0	0
Bradyrhizobium sp. III	2	1	1	1	0	1	0	0	0	1	0	0	0
Bradyrhizobium sp. IV	1	0	1	0	0	0	0	0	0	0	1	0	0
Bradyrhizobium sp. V	1	0	1	1	0	0	0	1	0	0	0	0	0
Bradyrhizobium sp. VI	25	0	25	12	13	0	0	12	13	0	0	0	0
Bradyrhizobium sp. VII	34	7	27	14	20	1	6	13	14	0	0	0	0
Bradyrhizobium sp. VIII	2	0	2	2	0	0	0	2	0	0	0	0	0
Bradyrhizobium sp. IX	2	0	2	2	0	0	0	2	0	0	0	0	0
Bradyrhizobium sp. X	1	0	1	0	1	0	0	0	1	0	0	0	0
Bradyrhizobium sp. XI	3	2	1	1	0	0	0	1	0	1	1	0	0

Bradyrhizobium sp. XII	14	9	5	3	2	0	2	3	0	2	1	4	2
Bradyrhizobium sp. XIII	1	0	1	0	1	0	0	0	1	0	0	0	0
Bradyrhizobium sp. XIV	1	1	0	0	1	0	1	0	0	0	0	0	0
Bradyrhizobium sp. XV	1	1	0	0	0	0	0	0	0	1	0	0	0
Bradyrhizobium sp. XVI	7	0	7	0	7	0	0	0	7	0	0	0	0
Bradyrhizobium sp. XVII	10	0	10	1	9	0	0	1	9	0	0	0	0
Bradyrhizobium sp. XVIII	1	1	0	1	0	1	0	0	0	0	0	0	0
Bradyrhizobium sp. XIX	27	9	18	11	16	1	8	10	8	0	0	0	0
Bradyrhizobium sp. XX	3	0	3	3	0	0	0	3	0	0	0	0	0
Bradyrhizobium sp. XXI	4	1	3	2	2	0	1	2	1	0	0	0	0
Bradyrhizobium sp. XXII	6	0	6	2	4	0	0	2	4	0	0	0	0
Bradyrhizobium sp. XXIII	1	0	1	1	0	0	0	1	0	0	0	0	0
Bradyrhizobium sp. XXIV	3	0	3	2	1	0	0	2	1	0	0	0	0
Bradyrhizobium sp. XXV	1	0	1	1	0	0	0	1	0	0	0	0	0
Bradyrhizobium sp. XXVI	1	0	1	1	0	0	0	1	0	0	0	0	0
Bradyrhizobium sp. XXVII	5	5	0	2	3	2	3	0	0	0	0	0	0
Bradyrhizobium sp. XXVIII	1	0	1	0	1	0	0	0	1	0	0	0	0
Bradyrhizobium sp. XXIX	76	0	76	37	39	0	0	37	39	0	0	0	0
Bradyrhizobium sp. XXX	1	0	1	0	1	0	0	0	1	0	0	0	0
Total	1001	562	439	381	355	180	170	201	185	84	57	61	63

[a]All, all samples; [b]NC, north China; [c]SC, south China.

Table S12 Alpha diversity indices for peanut rhizobium distributed in different regions or cultivars.

Region or cultivar	Shannon.Wiener	Simpson	*J*
All	2.36	0.86	0.63
North China	1.73	0.75	0.57
South China	2.54	0.88	0.71
X9-all	2.19	0.83	0.65
X9-north China	1.27	0.66	0.58
X9-south China	2.29	0.84	0.70
M6-all	2.41	0.88	0.74
M6-north China	1.70	0.75	0.68
M6-south China	2.41	0.88	0.78
H19	1.29	0.65	0.62
H30	1.83	0.80	0.79
H33	1.47	0.69	0.71
H29	0.99	0.57	0.71

Table S13 A multivariate test of MANOVA indicating the effect of environmental variables on peanut rhizobia obtained from different sampling sites and different peanut cultivars.

	Effect	Value	F	Hypothesis df	Error df	Sig.	Partial Eta Squared
Intercept	Pillai's Trace	0.996	1162.751	12.000	53.000	0.000	0.996
	Wilks' Lambda	0.004	1162.751	12.000	53.000	0.000	0.996
	Hotelling's Trace	263.264	1162.751	12.000	53.000	0.000	0.996
	Roy's Largest Root	263.264	1162.751	12.000	53.000	0.000	0.996
Genospecies	Pillai's Trace	4.423	0.934	480.000	768.000	0.794	0.369
	Wilks' Lambda	0.002	0.999	480.000	653.050	0.501	0.410
	Hotelling's Trace	10.075	1.074	480.000	614.000	0.203	0.456
	Roy's Largest Root	2.768	4.429	40.000	64.000	0.000	0.735
Peanut cultivar	Pillai's Trace	1.059	1.276	60.000	285.000	0.099	0.212
	Wilks' Lambda	0.166	1.965	60.000	251.957	0.000	0.302
	Hotelling's Trace	3.764	3.224	60.000	257.000	0.000	0.429
	Roy's Largest Root	3.425	16.269	12.000	57.000	0.000	0.774
Genospecies * Peanut cultivar	Pillai's Trace	2.161	0.370	456.000	768.000	1.000	0.180
	Wilks' Lambda	0.065	0.385	456.000	649.762	1.000	0.204
	Hotelling's Trace	3.713	0.417	456.000	614.000	1.000	0.236
	Roy's Largest Root	1.682	2.832	38.000	64.000	0.000	0.627

Table S14 Tests of between-subjects effects of MANOVA results indicating the effect of environmental variables on peanut rhizobia obtained from different sampling sites and peanut cultivar.

Source	Dependent variable	Type III sum of squares	df	Mean square	F	Sig.	Partial Eta squared
Corrected model	Isolates[a]	5776.709[a]	83	69.599	0.597	0.986	0.436
	AK	300235.078	83	3617.290	0.581	0.990	0.430
	AP	15085.067	83	181.748	0.570	0.992	0.425
	AN	86864.772	83	1046.564	0.870	0.727	0.530
	TN	0.504	83	0.006	0.894	0.687	0.537
	OC	70.405	83	0.848	0.719	0.922	0.482
	EC	156127.439	83	1881.053	0.726	0.915	0.485
	pH	115.756	83	1.395	1.233	0.192	0.615
	MAP	16931215.479	83	203990.548	1.105	0.340	0.589
	MAT	2010.277	83	24.220	1.026	0.461	0.571
	PS	4.147	83	0.050	0.961	0.571	0.555
	TS	39.214	83	0.472	0.309	1.000	0.286
Intercept	Isolates	1310.516	1	1310.516	11.234	0.001	0.149
	AK	4257093.959	1	4257093.959	683.319	0.000	0.914
	AP	36816.292	1	36816.292	115.476	0.000	.643
	AN	262308.240	1	262308.240	217.946	0.000	0.773
	TN	0.912	1	0.912	134.126	0.000	0.677
	OC	78.566	1	78.566	66.576	0.000	0.510
	EC	560861.255	1	560861.255	216.570	0.000	0.772
	pH	2269.089	1	2269.089	2005.596	0.000	0.969
	MAP	49961038.619	1	49961038.619	270.652	0.000	0.809
	MAT	10572.656	1	10572.656	447.812	0.000	0.875
	PS	29.800	1	29.800	573.303	0.000	0.900
	TS	24.591	1	24.591	16.072	0.000	0.201
Genospecies	Isolates	3401.752	40	85.044	0.729	0.857	0.313
	AK	200458.406	40	5011.460	0.804	0.768	0.335
	AP	8912.780	40	222.819	0.699	0.887	0.304
	AN	54829.592	40	1370.740	1.139	0.316	0.416
	TN	0.304	40	0.008	1.118	0.340	0.411
	OC	45.618	40	1.140	0.966	0.539	0.377
	EC	85324.192	40	2133.105	0.824	0.742	0.340
	pH	68.082	40	1.702	1.504	0.071	0.485
	MAP	9049136.867	40	226228.422	1.226	0.231	0.434
	MAT	888.341	40	22.209	0.941	0.576	0.370
	PS	2.280	40	0.057	1.097	0.365	0.407
	TS	11.036	40	0.276	0.180	1.000	0.101
Peanut cultivar	Isolates	296.009	5	59.202	0.507	0.770	0.038

	AK	27006.123	5	5401.225	0.867	0.508	0.063
	AP	1033.956	5	206.791	0.649	0.664	0.048
	AN	15881.390	5	3176.278	2.639	0.031	0.171
	TN	0.033	5	0.007	0.967	0.445	0.070
	OC	3.865	5	0.773	0.655	0.659	0.049
	EC	32759.919	5	6551.984	2.530	0.038	0.165
	pH	6.548	5	1.310	1.158	0.340	0.083
	MAP	732365.219	5	146473.044	0.793	0.558	0.058
	MAT	154.522	5	30.904	01.309	0.271	0.093
	PS	0.091	5	0.018	0.352	0.879	0.027
	TS	2.236	5	0.447	0.292	0.916	0.022
Genospecies *	Isolates	1518.911	38	39.971	0.343	1.000	0.169
peanut cultivar	AK	81058.610	38	2133.121	0.342	1.000	0.169
	AP	4642.373	38	122.168	0.383	0.999	0.185
	AN	16318.330	38	429.430	0.357	1.000	0.175
	TN	0.093	38	0.002	0.360	0.999	0.176
	OC	11.562	38	0.304	0.258	1.000	0.133
	EC	19422.709	38	511.124	0.197	1.000	0.105
	pH	33.147	38	0.872	0.771	0.805	0.314
	MAP	2932191.673	38	77162.939	0.418	0.998	0.199
	MAT	387.232	38	10.190	0.432	0.997	0.204
	PS	1.578	38	0.042	0.799	0.770	0.322
	TS	13.273	38	0.349	0.228	1.000	0.119
Error	Isolates	7466.014	64	116.656			
	AK	398721.312	64	6230.021			
	AP	20404.641	64	318.823			
	AN	77027.043	64	1203.548			
	TN	0.435	64	0.007			
	OC	75.526	64	1.180			
	EC	165744.103	64	2589.752			
	pH	72.408	64	1.131			
	MAP	11814084.764	64	184595.074			
	MAT	1511.013	64	23.610			
	PS	3.327	64	0.052			
	TS	97.927	64	1.530			
Total	Isolates	20013.000	148				
	AK	13042903.750	148				
	AP	173832.410	148				
	AN	1137189.140	148				
	TN	4.431	148				
	OC	466.539	148				
	EC	2263058.840	148				

	pH	6785.868	148
	MAP	192146382.000	148
	MAT	38584.020	148
	PS	94.470	148
	TS	220.391	148
Corrected total	Isolates	13242.723	147
	AK	698956.390	147
	AP	35489.707	147
	AN	163891.816	147
	TN	0.939	147
	OC	145.931	147
	EC	321871.542	147
	pH	188.164	147
	MAP	28745300.243	147
	MAT	3521.290	147
	PS	7.473	147
	TS	137.141	147

[a], the isolate number of the corresponding genospecies.
